# A nanoscale metal organic frameworks-based vaccine synergises with PD-1 blockade to potentiate anti-tumour immunity

**DOI:** 10.1038/s41467-020-17637-z

**Published:** 2020-07-31

**Authors:** Xia Li, Xiupeng Wang, Atsuo Ito, Noriko M. Tsuji

**Affiliations:** 1 Department of Life Science and Biotechnology, Health and Medical Research Institute, National Institute of Advanced Industrial Science and Technology (AIST), Central 6, 1-1-1 Higashi, Tsukuba, Ibaraki 305-8566 Japan; 2 Department of Life Science and Biotechnology, Cellular and Molecular Biotechnology Research Institute, National Institute of Advanced Industrial Science and Technology (AIST), Central 6, 1-1-1 Higashi, Tsukuba, Ibaraki 305-8566 Japan

**Keywords:** Cancer, Immunology, Oncology, Chemistry

## Abstract

Checkpoint blockade therapy has provided noteworthy benefits in multiple cancers in recent years; however, its clinical benefits remain confined to 10–40% of patients with extremely high costs. Here, we design an ultrafast, low-temperature, and universal self-assembly route to integrate immunology-associated large molecules into metal-organic-framework (MOF)-gated mesoporous silica (MS) as cancer vaccines. Core MS nanoparticles, acting as an intrinsic immunopotentiator, provide the niche, void, and space to accommodate antigens, soluble immunopotentiators, and so on, whereas the MOF gatekeeper protects the interiors from robust and off-target release. A combination of MOF-gated MS cancer vaccines with systemic programmed cell death 1 (PD-1) blockade therapy generates synergistic effects that potentiate antitumour immunity and reduce the effective dose of an anti-PD-1 antibody to as low as 1/10 of that for PD-1 blockade monotherapy in E.G7-OVA tumour-bearing mice, with eliciting the robust adaptive OVA-specific CD8^+^ T-cell responses, reversing the immunosuppressive pathway and inducing durable tumour suppression.

## Abstract (web summary)

Nanoparticle-based strategies have been proposed to enhance the benefit of cancer immunotherapy. Here the authors show that a cancer vaccine based on metal organic frameworks-gated mesoporous silica nanoparticles for antigen and immune potentiators delivery boosts the therapeutic efficacy of low-dose anti-PD1.

## Introduction

The clinical benefits of checkpoint blockade therapy rekindle the hope of cancer immunotherapy^1–5^. However, objective response rates in checkpoint blockade therapy targeting programmed cell death 1 (PD-1), cytotoxic T lymphocyte-associated protein-4 (CTLA4) or programmed cell death ligand 1 (PD-L1) remain at ~10–40% owing to multiple immunosuppressive factors, such as T-cell exclusion, immunosuppressive cells, deprivation of tumour-infiltrating lymphocytes and neoantigens, and negatively regulating markers and cytokines^1–5^. On the other hand, checkpoint blockade therapy is associated with significantly high costs that greatly imposes economic burden on the patients and society^6^. Most importantly, checkpoint blockade with systemic administration of antibodies (Abs) is associated with the risk of immune-related adverse events including cytokine storm and autoimmune diseases in the long-term^1,2,7,8^.

To broaden the clinical benefit and minimise the therapeutic costs, the use of a combination cancer immunotherapy is considered to be the future direction of cancer treatment^3,5,9–14^. Combination cancer immunotherapy that simultaneously “releases the immunological break” using immune checkpoint inhibitors and “presses the immunological accelerator” by stimulating antigen presentation, which thus prime and activate effector T-cell responses, would be more effective than monotherapy^3,5,9–13^. That is, appropriate cancer vaccines that stimulate antigen presentation and T-cell priming when given in combination with immune checkpoint inhibitors are expected to minimise the dose, therapeutic costs and the risk of adverse events induced by immune checkpoint inhibitors. Since the response rate in checkpoint blockade therapy depends on T-cell immunity^15^, a combination of cancer vaccines may increase the response rate by strengthening the immunogenicity of cancer antigens, triggering and amplifying the specific T-cell immune responses towards cancer antigens^9–13,15–18^.

For successful cancer vaccines, a rational design of adjuvants to integrate cancer antigens and immunopotentiators is pivotal, since the administration of these components separately may result in nonspecific immune responses in the entire body and severe side effects^19^. A majority of cancer vaccines adopt a mixture of cancer antigens and immunopotentiators with or without vehicles^9–13,15,16^. However, they have the problems of initial burst and off-target release, resulting in a decrease in vaccination efficiency. To realise the full efficacy of cancer vaccines, the requirements indispensable for adjuvants include (1) efficient encapsulation of cancer antigens and immunopotentiators to prevent their initial burst release and realise their controlled release, (2) targeting delivery to antigen-presenting cells (APCs) and lymph nodes, (3) shaping effective anti-tumour T-cell responses, and (4) good biocompatibility. Satisficing all these requirements is not easy. Inspired by the superior biomimetic mineralisation encapsulation capability of the metal organic framework (MOF) for biomolecules^20–22^ and excellent intrinsic immune-shaping properties of mesoporous silica (MS)^23,24^, we fabricated nanoadjuvants on the basis of MOF-gated MS (MS@MOF) to realise targeted delivery to APCs and lymph nodes, and navigate antitumour immunity.

Here, we propose an ultrafast, low-temperature, universal self-assembly route to integrate a cancer antigen and an immunopotentiator into each nanoparticle consisting of MS as a core container and MOF as a gatekeeper for fabricating MS@MOF cancer vaccines as the magic bullet for combination cancer immunotherapy. An integrated formulation of cancer vaccines with a pH-switch button can realise targeted, controlled and efficient codelivery of the antigen (ovalbumin, OVA) and immunopotentiator (polyinosinic-polycytidylic acid, polyIC) to draining lymph node, enhance their availability and minimise the off-target effects. Furthermore, MS@MOF cancer vaccines, in combination with systemic checkpoint blockade at merely 10% dose of PD-1 blockade monotherapy^9,11,25^, exhibit synergetic effects that reverse the immunosuppressive tumour microenvironment, elicit robust adaptive cancer antigen-specific immune responses, and effectively induce durable tumour suppression in tumour-bearing mice.

## Results

### Hierarchical self-assembling synthesis of MS@MOF nanoadjuvants

**Fig. 1:**
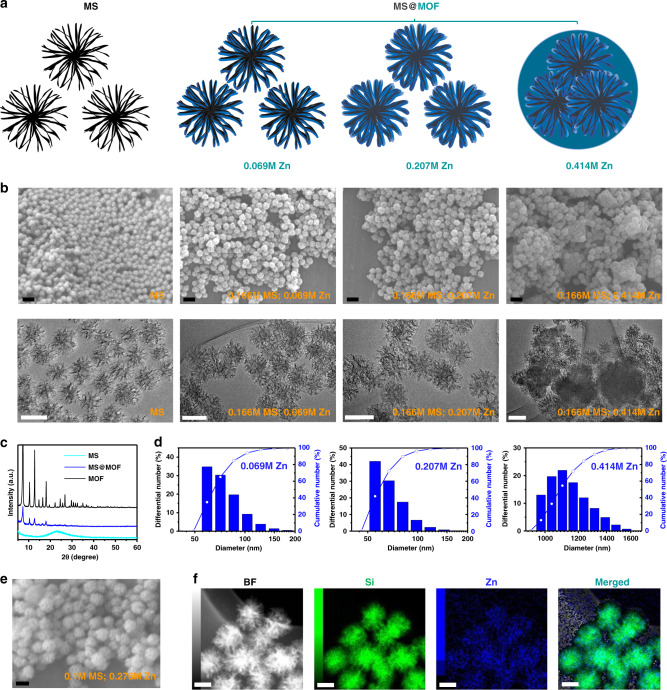
Hierarchical self-assembling synthesis of MOF-gated MS nanoadjuvants. **a** Scheme of MS and MOF-gated MS nanostructure, **b** SEM and TEM images of MS and MOF-gated MS with different ratio of MS/MOF (0.166 M MS, 0.069 M Zn^2+^; 0.166 M MS, 0.207 M Zn^2+^; and 0.166 M MS, 0.414 M Zn^2+^). Scale bars, 200 nm (upper, SEM); 100 nm (down, TEM). **c** XRD patterns of MS, MOF-gated MS and MOF. **d** Particle size distribution of MOF-gated MS with different ratio of MS/MOF (0.166 M MS, 0.069 M Zn^2+^; 0.166 M MS, 0.207 M Zn^2+^; and 0.166 M MS, 0.414 M Zn^2+^). **e** SEM image and **f** STEM mapping of MOF-gated MS (synthesis parameter: 0.1 M MS, 0.276 M Zn^2+^). Scale bar in **e**, 100 nm. Scale bar in **f**, 50 nm.

MS@MOF free of antigens and molecular immunopotentiators at various MS-to-MOF ratios were synthesised by immersing MS in solutions containing Zn^2+^ and 2-methylimidazole at 0 °C for 15 min (Fig. 1; Supplementary Figs. 1–4). MS exhibits stellated pore channels and a dendritically open gate up to 35 nm (Supplementary Fig. 1a, b). When the Zn^2+^ and 2-methylimidazole concentrations are low, MOF precipitate onto the inner wall of stellated pore channels in MS and form a thin layer. With increasing Zn^2+^ and 2-methylimidazole concentrations or decreasing MS concentration, the precipitated MOF gradually increases in amount, fills the pore channels and blocks the open gate. Extremely high Zn^2+^ and 2-methylimidazole concentration solutions or low MS initial amounts result in the aggregation of MS nanoparticles into one large particle (Fig. 1a, b). Scanning electron microscopy (SEM) images show that with increasing MOF amount, MS@MOF gradually changes in shape from isolated nanospheres of ~100 nm to aggregated nanocomplexes of ~500 nm (Fig. 1b; Supplementary Figs. 2 and 3). Wide-angle X-ray diffraction (WAXRD) patterns exhibit the crystallinity of ZIF-8 MOF phase in the MS@MOF with the intense diffraction peaks at around 7.3, 10.4, 12.7, 14.6, 16.4 and 18.0°, compared with the amorphous silica phase in MS with a broad peak at ~23° (Fig. 1c). The hydrodynamic diameter of MS@MOF increases from 100 to 1200 nm with the increase in MOF amount, as determined by dynamic light scattering (DLS; Fig. 1d). The zeta potential of MS@MOF is centred at −18 mV in phosphate-buffered saline [PBS(−)], whereas those of MS and MOF are at around −16 and −24 mV, respectively (Supplementary Fig. 4a). The specific surface areas of MS, MS@MOF and MOF are 526, 323 and 1098 m^2^/g, respectively (Supplementary Fig. 4b–d). The average pore size of MS is 13.2 nm, which is large enough for adsorption of biomolecules. In contrast, MOF shows a small average pore size of about 1.6 nm, which makes it useful as the gatekeeper to protect the interiors from robust release. Scanning transmission electron microscopy (STEM) mapping shows the uniform distribution of Zn^2+^ elements in the entirely emanative channels of MS from the exterior to the interior (Fig. 1f).

MOF-gated MOF (MOF@MOF) was also fabricated as a control (Supplementary Figs. 5 and 6). First, the MOF core of ~100–200 nm was prepared using Zn^2+^ (0.69 M) and 2-methylimidazole (3.13 M) solutions. In the second step, the MOF@MOF was formed by immersing the MOF core in Zn^2+^ (0.138, 0.276 and 0.345 M) and 2-methylimidazole (0.626, 1.252 and 1.565 M) solutions. By varying the Zn^2+^ and 2-methylimidazole concentrations in the second step, we can correspondingly adjust the shell thickness and aggregation status of the obtained MOF@MOF.

### MOF-gated MS encapsulating model antigens and molecular immunopotentiators

The MOF-gated MS encapsulated and immobilised model antigens and molecular immunopotentiators into the open pore channels of MS owing to the overlaying growth of MOF at a low temperature on MS. First, the encapsulation capability of MOF was investigated by simply supplementing OVA and polyIC into Zn^2+^ and 2-methylimidazole aqueous solutions (Supplementary Figs. 7–12). OVA was efficiently encapsulated into MOF (OVA*in*MOF). Increasing the OVA concentration from 0 to 25 mg/mL did not affect the particle size of the formed MOF maintaining a size in the range of 100–200 nm. XRD patterns of OVA*in*MOF with various OVA concentrations exhibit the ZIF-8 phase. The encapsulation efficiency of OVA within MOF was ~75% when the OVA concentration was 25 mg/mL. Moreover, polyIC was encapsulated into MOF to obtain polyIC*in*MOF, which exhibits a particle size in the range of 100–200 nm and XRD patterns of the ZIF-8 phase, being similar to those of OVA*in*MOF. The encapsulation efficiencies of polyIC within MOF at 25 and 5 mg/mL of polyIC were about 55 and 80%, respectively.

Then, versatile biomolecules (model antigen, molecular immunopotentiator and checkpoint inhibitor antibodies) were encapsulated in the open pore channels of MS in conjunction with the growth of MOF on MS by hierarchical self-assembly to fabricate MOF-gated MS cancer vaccines. In a typical synthesis, well-dispersed MS nanoparticles were initially immersed in an OVA-containing aqueous solution to adsorb OVA sufficiently, and Zn^2+^ and 2-methylimidazole were added to the solution to form inner OVA*in*MOF within the stellated channels of MS, named as MS@(OVA*in*MOF). In the second step, the obtained MS@(OVA*in*MOF) particles were immersed in an aqueous solution containing either an anti-CTLA4 Ab or polyIC, Zn^2+^ and 2-methylimidazole to fabricate (MS@OVA*in*MOF)@(anti-CTLA4*in*MOF) or (MS@OVA*in*MOF)@(polyIC*in*MOF). The encapsulation efficiency of the anti-CTLA4 Ab within MOF-gated MS was calculated to be about 100% from the standard curve (Supplementary Fig. 13). The zeta potentials of OVA, anti-CTLA4 Ab and polyIC in PBS(−) were about −9.5, −2.5 and −27 mV, respectively (Supplementary Fig. 14). The zeta potential of MS@(OVA*in*MOF) was ~−16 mV. After the second step, the zeta potentials of (MS@OVA*in*MOF)@(anti-CTLA4*in*MOF) and (MS@OVA*in*MOF)@(polyIC*in*MOF) shifted to about −10 and −25 mV, respectively. The changes in the zeta potential reflect the successful encapsulation of various biomolecules into the MOF-gated MS.

We evaluated reproducibility of the manufacturing method for different batches by XRD, SEM and TEM analyses (Supplementary Fig. 15). The results show that the morphology and particle size of samples from different batches were highly uniform without obvious difference. MS@(OVA*in*MOF), (MS@OVA*in*MOF)@(polyIC*in*MOF) and (MS@OVA*in*MOF)@(anti-CTLA4*in*MOF) synthesised from different batches exhibit similar ZIF-8 MOF phases with the intense diffraction peaks at around 7.3, 10.4, 12.7, 14.6, 16.4 and 18.0° in the WAXRD patterns. MS@(OVA*in*MOF), (MS@OVA*in*MOF)@(polyIC*in*MOF), and (MS@OVA*in*MOF)@(anti-CTLA4*in*MOF) show similar nanosphere morphologies and sizes of ~100 nm.

The presence of the agents in the nanoadjuvants was further confirmed by sodium dodecyl sulfate-polyacrylamide gel electrophoresis (SDS–PAGE, Supplementary Fig. 16). OVA-loaded nanoadjuvants and corresponding supernatant samples after suspending them in water, including free OVA, OVA*on*MS, MS@(OVA*in*MOF) and OVA*in*MOF, were tested. Herein, OVA solution was mixed with MS to prepare OVA*on*MS. For the supernatants of free OVA, the band of OVA was clearly detected. For the supernatants of OVA*on*MS, the band of OVA becomes weaker owing to the partial desorption of OVA molecules from MS. In contrast, no obvious band of OVA was detected in the SDS–PAGE for the supernatants of MS@(OVA*in*MOF) and OVA*in*MOF, indicating a strong affinity between OVA and the carriers (Supplementary Fig. 16a). Moreover, the OVA-loaded nanoadjuvants, including OVA*on*MS, MS@(OVA*in*MOF), and OVA*in*MOF, show the band of OVA clearly in the SDS–PAGE similarly to the free OVA group, indicating the presence of OVA in the nanoadjuvants (Supplementary Fig. 16b).

We developed an ultrafast, low-temperature, universal aqueous-phase route to encapsulate high-molecular-weight cancer antigens and immunopotentiators into stellated pore channels of MS in conjunction with the low-temperature growth of MOF in an economically and highly efficient way. The open and stellated pore channels with a size as large as 35 nm in MS and the subsequent crystallisation of MOF entrapping biomolecules provide the possibility to accommodate and encapsulate a wide range of high-molecular-weight biomolecules into the MOF-gated MS. Although multiple biomolecules were encapsulated in one particle of MOF-gated MS by the layer-by-layer self-assembly process, they can also be encapsulated by a one-pot route. The present synthesis techniques are quite essential for cancer vaccines since the use of a combination of various biomolecules is crucial to eradicating established tumours. This MOF-gated strategy applies to not only MS nanoparticles, but also MS scaffolds and other nanomaterials.

### PH-sensitive degradation and biomolecule release from MOF-gated MS

**Fig. 2:**
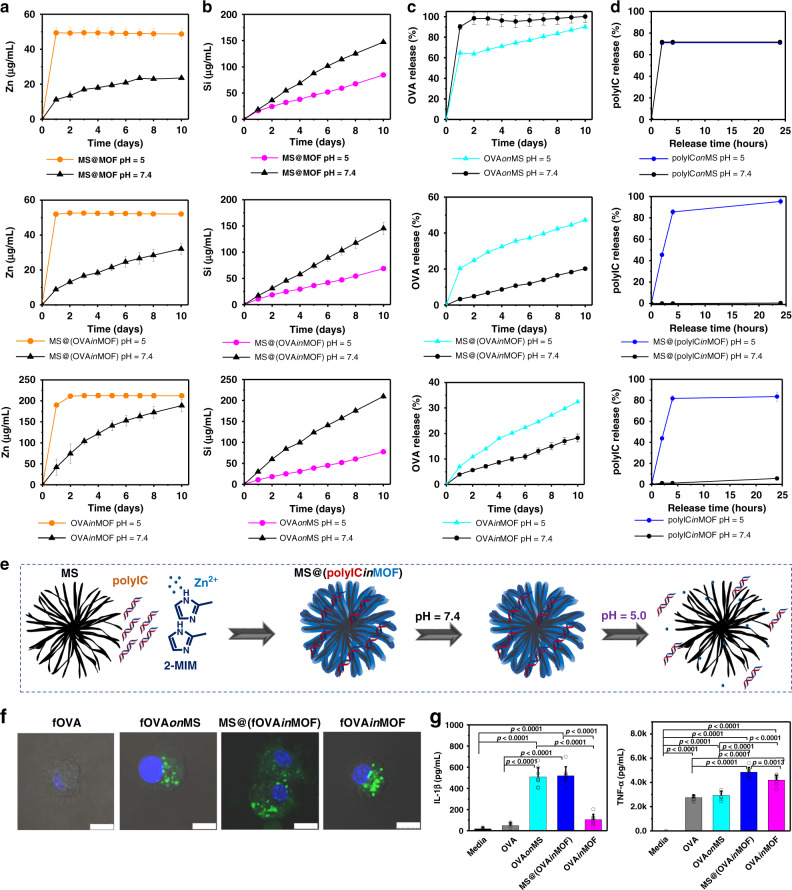
Degradation and biomolecule release of nanoadjuvants, and BMDCs activation in vitro. **a**, **b** pH-responsive degradation of MS, MOF-gated MS and MOF (*n* = 3 independent samples); **c** OVA release from OVA*on*MS, MS@(OVA*in*MOF) and OVA*in*MOF at different pH values (*n* = 4 independent samples); **d** polyIC release from polyIC*on*MS, MS@(polyIC*in*MOF) and polyIC*in*MOF at different pH values (*n* = 3 independent samples). **e** Scheme of MS@(polyIC*in*MOF) fabrication and its release under different pH values. **f** Confocal laser scanning microscopic images of the cellular uptake of free fOVA, fOVA*on*MS, MS@(fOVA*in*MOF) and fOVA*in*MOF by BMDCs. Hoechst, blue; fOVA (model cancer antigen), green. Scale bars, 10 μm. **g** IL-1β and TNF-α secreted by BMDCs when culturing with different nanoadjuvants at day 1 (*n* = 8 independent samples, one-way ANOVA followed by Tukey’s multiple comparisons post hoc test; IL-1β, *p* < 0.0001; TNF-α, *p* < 0.0001). All data in **a**–**d** are presented as mean ± S.D. Data in **g** are presented as mean + S.D.

The degradability of nanoadjuvants is a key parameter to be considered for future clinical applications. Here, we comprehensively evaluated the degradation properties and biomolecule release properties of MOF-gated MS (Fig. 2a–e; Supplementary Figs. 17–21). MS gradually degrades into silicic acid over time, whereas MOF degrades into Zn ions and imidazolate. The degradation profiles of MS@(OVA*in*MOF) or MS@MOF were studied by inductively coupled plasma atomic emission spectroscopy (ICP-AES) in acetate buffer (pH  = 5) and Tris-HCl buffer (pH = 7.4). The degradation properties of OVA*in*MOF and OVA*on*MS were also investigated as the controls. In neutral buffer, MS@MOF exhibited a slow and sustained release of Zn ions with a low initial release rate up to ~11 μg/mL within 1 day followed by a cumulative release rate of up to about 23 μg/mL within 8 days. On the other hand, in acetate buffer, a burst release of Zn ions up to about 49 μg/mL was observed within 1 day. MS@(OVA*in*MOF) and OVA*in*MOF showed a similar trend in Zn release to MS@MOF, although OVA*in*MOF exhibits a faster Zn release in neutral buffer than the other two. MS@MOF, MS@(OVA*in*MOF) and OVA*on*MS demonstrate a sustained release of Si ions in both neutral and acetate buffers, although the Si release in neutral buffer is faster than that in acetate buffer. In addition, Tris-HCl buffer supplemented with 10% serum was also used to test the degradation behaviours of MOF-gated MS. As a whole, the degradation curves in serum-supplemented buffer are similar to those in pure buffer (Supplementary Fig. 20a, b).

The release of biomolecules from MOF-gated MS shows the same trend as the degradation of MOF-gated MS. OVA*in*MOF and MS@(OVA*in*MOF) exhibit a slow and sustained release of OVA in neutral buffer, whereas they show a burst release of OVA in acetate buffer (Fig. 2c, Supplementary Figs. 17 and 18). Similarly, polyIC*in*MOF and MS@(polyIC*in*MOF) exhibit a slow and sustained release of polyIC in neutral buffer, and a burst release of polyIC in acetate buffer (Fig. 2d, Supplementary Fig. 19). OVA*on*MS and polyIC*on*MS show a burst release of OVA or polyIC in both neutral and acetate buffers (Fig. 2c, d). The presence of serum in the buffer did not markedly affect the release of biomolecules from MOF-gated MS (Fig. 2c, d; Supplementary Fig. 20c, d). Here, ferritin was used instead of OVA to investigate protein release in serum. In Tris-HCl buffer supplemented with 10% serum, ferritin*in*MOF, polyIC*in*MOF, MS@(ferritin*in*MOF) and MS@(polyIC*in*MOF) exhibit a slow and sustained release of ferritin or polyIC, whereas ferritin*on*MS and polyIC*on*MS show a burst release.

The slow degradation in neutral buffer and the burst degradation in acetate buffer of MOF-gated MS is advantageous for preventing the premature release of antigens and immunopotentiators in the extracellular environment and facilitating their delivery into the intracellular environment. MOF-gated MS encapsulating biomolecules exhibit the pH-responsive release of biomolecules with a slow release in neutral buffer and a rapid release in acetate buffer. In addition, the coordination of Zn with OVA enhances the retention of OVA in MS@(OVA*in*MOF) or OVA*in*MOF, which accounts for the slower release of OVA than of other molecules. The controlled degradability and release of MOF-gated MS in a pH-responsive manner can facilitate antigen delivery, antigen presentation, and priming antitumour T-cell immunity. The pH-responsive endosomolytic nanoadjuvants facilitate the antigen escape from endo/lysosomes to the cytoplasm and the subsequent cross-presentation associated with major histocompatibility complex (MHC) I molecules^26^. Here, MOF-gated MS exhibits a pH-responsive release of biomolecules with a slow release in neutral buffer and a rapid release in acidic buffer, which suggests the potential of vaccination based on MOF-gated MS to enhance cross-presentation of cancer antigens to CD8^+^ T cells.

### Cellular uptake, activation and antigen presentation of dendritic cells in vitro

We first examined the impact of MOF-gated MS on cellular uptake and activation of APCs, since the activation of APCs is the first step to initiate adaptive immune responses. Here, fluorescein-conjugated OVA (fOVA) was used as model antigen. Bone marrow dendritic cells (BMDCs) were cultured with fOVA*on*MS, MS@(fOVA*in*MOF), and fOVA*in*MOF using FITC-conjugated OVA (fOVA) or the counterparts of OVA to investigate the effects of nanoadjuvants on cellular uptake and activation of BMDCs in vitro (Fig. 2f, g; Supplementary Figs. 22–24). The medium, free fOVA and OVA were used as the controls. The free fOVA group shows very weak green fluorescence from FITC, whereas the fOVA*on*MS, MS@(fOVA*in*MOF), and fOVA*in*MOF groups show intense green fluorescence. The average fluorescence intensities of BMDCs after coculture with free fOVA, fOVA*on*MS, MS@(fOVA*in*MOF) and fOVA*in*MOF are 47, 4594, 14383 and 8956, respectively (Fig. 2f, Supplementary Fig. 22). The presence of MS, MS@MOF or MOF facilitates the secretion of interleukin (IL)-1β and tumour necrosis factor (TNF)-α from BMDCs (Fig. 2g). The OVA*on*MS and MS@(OVA*in*MOF) stimulate much more IL-1β secretion from BMDCs than the free OVA and OVA*in*MOF. MS@(OVA*in*MOF) stimulates more TNF-α secretion from BMDCs than OVA, OVA*on*MS and OVA*in*MOF. BMDCs cocultured with MS@(OVA*in*MOF) show significantly increased MHC-I^+^, MHC-II^+^, CD80^+^, CD40^+^, and CCR7^+^ cell populations as compared with those cultured with free OVA. BMDCs cocultured with MS@(OVA*in*MOF) show the highest MHC-I^+^, MHC-II^+^, CD80^+^, and CCR7^+^ cell populations among all the groups. BMDCs cocultured with OVA*in*MOF show higher CD40^+^ cell populations than those cultured with free OVA, OVA*on*MS, and MS@(OVA*in*MOF) (Supplementary Figs. 23 and 24). MHC I and MHC II molecules on the surface of APCs mediate antigen presentation. The cross-presentation of exogenous antigens on MHC I molecules is necessary for priming CD8^+^ T-cell responses and plays vital roles in antitumour immunity^18,27^. The chemokine receptor CCR7 plays a central role in mediating APC homing to lymph nodes. MOF-gated MS most efficiently enhances cancer antigen presentation, upregulates the expression of costimulatory molecules (CD40 and CD80), and promotes chemokine receptor CCR7 expression, which implies their great potential in cancer vaccines, compared with MS or MOF.

To investigate the effects of the mode of biomolecule loading on BMDCs activation, fOVA- or OVA- adsorbing MOF (MOF-ad) and fOVA- or OVA- encapsulating MOF by coprecipitation (MOF-en) were compared (Supplementary Figs. 25 and 26). The MOF-en group exhibits much higher cellular uptake of fOVA and higher secretion of TNF-α and IL-1β from BMDCs than the MOF-ad group. These results suggest that encapsulation of biomolecules into MOF by coprecipitation is superior to the simple adsorption of biomolecules onto the MOF surface. This finding further supports the above-mentioned results that MOF-gated MS exhibits much higher BMDC stimulation capability than MS.

### Prolonged retention, enhanced delivery to lymph nodes and promoted cross-presentation of antigens by MOF-gated MS in vivo

**Fig. 3:**
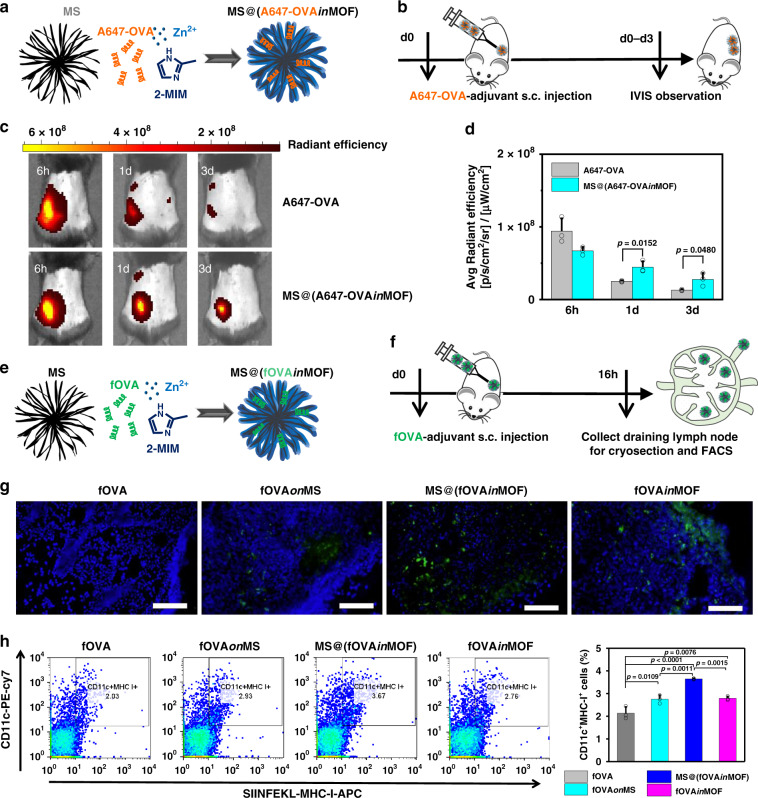
Antigen retention at vaccination site and delivery to lymph nodes. **a** Scheme of MS@(A647-OVA*in*MOF) fabrication. **b** Timeline of the injection of free A647-OVA and MS@(A647-OVA*in*MOF), and IVIS observation. **c** Representative images of A647-OVA model cancer antigen residues at the injection sites observed by IVIS fluorescent imaging system for free A647-OVA (upper) and MS@(A647-OVA*in*MOF) (lower). **d** Quantitative analysis of A647-OVA fluorescent intensity at the injection site at 6 h, 1 day and 3 day (*n* = 3 independent animals; Student’s *t* test, two-tailed). **e** Scheme of MS@(fOVA*in*MOF) fabrication. **f** Timeline of the injection of free fOVA and fOVA-loaded nanoadjuvants and subsequent collection of lymph nodes for cryosection and flow cytometry analysis. **g** Representative cryosection images of lymph nodes in mice vaccinated with: fOVA, fOVA*on*MS, MS@(fOVA*in*MOF) and fOVA*in*MOF. DAPI, blue; fOVA, green. Scale bars, 50 μm. **h** Representative flow cytometry plots (left) and populations (right) of CD11c^+^MHC-I^+^ dendritic cells with antigen cross-presentation in lymph nodes in mice vaccinated with: fOVA, fOVA*on*MS, MS@(fOVA*in*MOF) and fOVA*in*MOF (*n* = 3 independent samples, one-way ANOVA followed by Tukey’s multiple comparisons post hoc test, *p* < 0.0001). All data (**d**; **h**, right panel) are presented as mean + S.D.

We next investigated whether cancer antigens could be retained for a long time using Alexa Fluor 647-conjugated OVA (A647-OVA) as the model cancer antigen (Fig. 3a–d). The average fluorescence intensities of the free A647-OVA and MS@(A647-OVA*in*MOF) groups at the injection site are comparable at 6 h. The MS@(A647-OVA*in*MOF) group tends to show an average fluorescence intensity two times higher than free A647-OVA at 1 and 3 days.

To analyse the ability of APCs to capture and transport cancer antigens to the draining lymph nodes, MOF-gated MS loaded with fOVA was subcutaneously injected, and then, cryosections of the draining lymph nodes were observed 16 h after injection (Fig. 3e–g; Supplementary Fig. 27). The free fOVA group was used as the control. The green fluorescence intensities in the cryosections of lymph nodes are much higher for the fOVA*on*MS, MS@(fOVA*in*MOF) and fOVA*in*MOF groups than for the free fOVA group. To analyse the distribution of MOF-gated MS in vivo, MS@(A647-OVA*in*MOF) in organs of mice was examined by IVIS and ICP-AES analyse (Supplementary Fig. 28). From the IVIS images, MS@(A647-OVA*in*MOF) is mostly accumulated in nearby draining lymph nodes, whereas its amount is negligible in other organs, including the spleen, lung, heart, kidney, and liver. Mice administrated with MS@(A647-OVA*in*MOF) show significant increases in average fluorescence intensity, Si content, and Zn content in nearby draining lymph nodes as compared with control. Furthermore, cross-presentation of the OVA epitope on MHC I of DCs was analysed by flow cytometry using an anti-mouse OVA257-264 (SIINFEKL) peptide bound to H-2Kb. The nanoparticulate formulation of fOVA*on*MS, MS@(fOVA*in*MOF), or fOVA*in*MOF induces in the generation of larger numbers of CD11c^+^ MHC-I^+^ DCs in the lymph nodes than free fOVA. The MS@(fOVA*in*MOF) shows the highest efficiency of antigen cross-presentation among all the groups (Fig. 3h).

The effective adaptive antitumour immune response relies on the persistence of a cancer antigen in the distal injection site, the timely immune cell communication between the periphery and the draining lymph nodes, and the subsequent antigen presentation to T cells in lymph nodes^28–32^. Prolonged retention of antigens within adjuvants in the injection sites is considered to be crucial to ensuring the long-term stimulation of DCs to break immune tolerance^28–33^. Here, A647-OVA encapsulated within MOF-gated MS is retained for a significantly longer time around the injection site than free A647-OVA (Fig. 3a–d); thus, A647-OVA encapsulated within MOF-gated MS facilitates the long-term stimulation and activation of DCs and breaks the immune tolerance towards cancer antigens^16,28–33^.

### Prophylactic vaccines in xenografts

**Fig. 4:**
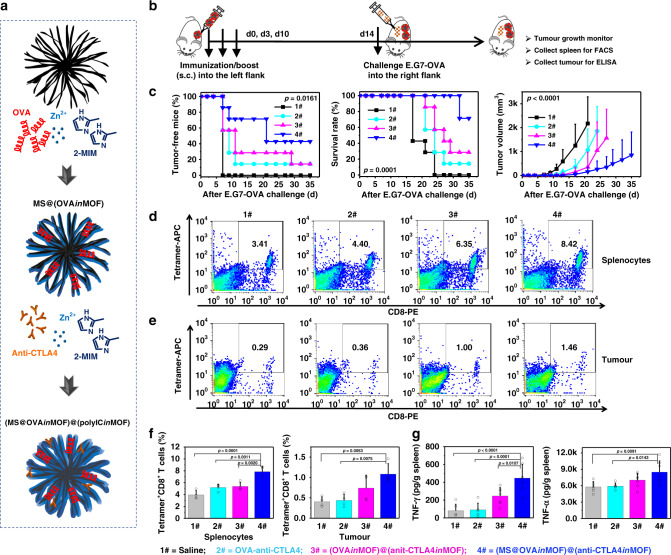
Antitumour activity and tumour-specific immune response in prophylactic mouse model. **a** Scheme of (MS@OVA*in*MOF)@(anti-CTLA4*in*MOF) fabrication. **b** Timeline of experiment: female C57Bl/6J mice were immunised with subcutaneous injection of cancer vaccines into the left flank three times at 0, 3 and 10 days, challenged with 5 × 10^5^ cells mouse^−1^ of E.G7-OVA cells into the right flank at 14 day and monitored whether vaccines inhibited tumour growth or not. 1# Saline group; 2# free OVA-anti-CTLA4 group (OVA, 100 μg/mouse; anti-CTLA4 Ab, 20 μg/mouse); 3# (OVA*in*MOF)@(anit-CTLA4*in*MOF) group (OVA, 100 μg/mouse; anti-CTLA4 Ab, 20 μg/mouse; MOF, 600 μg/mouse); and 4# (MS@OVA*in*MOF)@(anti-CTLA4*in*MOF) (OVA, 100 μg/mouse; anti-CTLA4 Ab, 20 μg/mouse; MS@MOF, 600 μg/mouse). **c** (MS@OVA*in*MOF)@(anti-CTLA4*in*MOF) prevents the tumour occurrence, prolongs the survival rate and suppresses the tumour growth. Kaplan–Meier curve of tumour-free mice (left) and mice survival (middle), and tumour volume curve (right) after subcutaneously challenging E.G7-OVA cells into the right flank of vaccinated mice (*n* = 7 independent animals; left, middle, log-rank; right, two-way ANOVA). **d**–**f** Representative flow cytometry plots (**d**, **e**) and population (**f**) of Tetramer^+^CD8^+^ T cells in splenocytes and tumour sites at the endpoint (*n* = 4 independent animals, one-way ANOVA followed by Tukey’s multiple comparisons post hoc test; splenocytes, *p* < 0.0001; tumour, *p* = 0.0036). **g** IFN-γ and TNF-α contents in the spleen (1#, 2#, *n* = 6 independent animals; 3#, 4#, *n* = 7 independent animals; one-way ANOVA followed by Tukey’s multiple comparisons post hoc test; IFN-γ, *p* < 0.0001; TNF-α, *p* = 0.0058). All data (**c**, right panel; **f, g**) are presented as mean + S.D.

To evaluate the effects of MOF-gated MS in a prophylactic tumour model, (MS@OVA*in*MOF)@(anti-CTLA4*in*MOF) cancer vaccines, comprising the cancer model antigen (OVA) and anti-CTLA4 Ab, were fabricated to check the antitumour efficacy using E.G7-OVA lymphoma (Fig. 4; Supplementary Fig. 29). Mice immunised with (MS@OVA*in*MOF)@(anti-CTLA4*in*MOF) show a higher ratio of tumour-free mice, significantly higher survival rate, and significantly smaller tumour volume than those administrated with (OVA*in*MOF)@(anit-CTLA4*in*MOF), free OVA-anti-CTLA4, and only saline. In the (MS@OVA*in*MOF)@(anti-CTLA4*in*MOF), (OVA*in*MOF)@(anit-CTLA4*in*MOF) and free OVA-anti-CTLA4 groups, the dose of anti-CTLA4 Ab (20 μg/mouse) was as low as 1/10 conventional dose. Mice immunised with (MS@OVA*in*MOF)@(anti-CTLA4*in*MOF) show the highest population of tetramer^+^CD8^+^ T cells in the spleen and tumour sites and the highest cytokine contents in the spleen (IFN-γ and TNF-α) among all the groups (Fig. 4). Notably, comparisons between free OVA-anti-CTLA4 and (OVA*in*MOF)@(anit-CTLA4*in*MOF; Fig. 4), or between free OVA and OVA*in*MOF (Supplementary Fig. 30) show that only MOF has no significant improvement in progression-free survival, overall survival or tumour volume.

In this study, MOF-gated MS induces higher antitumour efficacy than MOF adjuvant free of MS with an equivalent weight, which suggests that MS is a determining factor for stimulating antitumour immunity^24^. MS triggers antitumour immunity, which is derived from not only its functions as the carrier of antigens and immunopotentors but also its intrinsic immunomodulatory effects^23,24^. It should be mentioned here that the MS used in this study is quite different from the previously reported hollow MS^23^ in pore size (several tens nm and 3–6 nm in the present and previous MS, respectively) and particle morphology, resulting in clearly different characteristics in retention and release of biomolecules.

### Combination immunotherapy of MOF-gated MS vaccination and PD-1 blockade

**Fig. 5:**
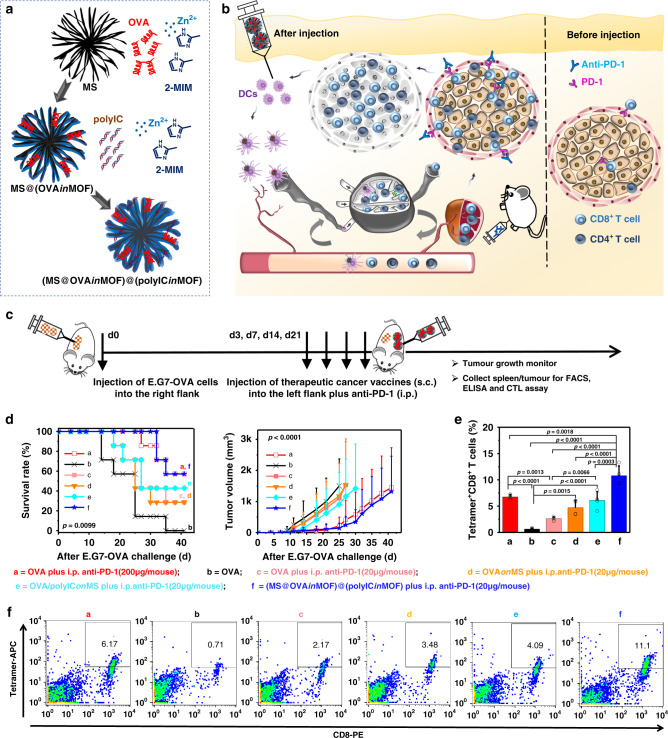
Antitumour activity and tumour-specific immune response in therapeutic mouse model. **a** Scheme of (MS@OVA*in*MOF)@(polyIC*in*MOF) fabrication. **b** Scheme of using MOF-gated MS cancer vaccine in combination with anti-PD-1 Ab to stimulate DCs cells activation, enhance cancer antigen – specific T-cell activity in lymph nodes and spleen, release immunological break and inhibit tumour growth. **c** Timeline of treatment: female C57Bl/6J mice were subcutaneously injected with 2 × 10^5^ cells mouse^−1^ of E.G7-OVA cells to establish tumours; On day 3, 7, 14, 21 post-tumour inoculation, mice were administrated with subcutaneous injection of cancer vaccines plus intraperitoneal injection of anti-PD-1 Ab; Tumour growth was continuously monitored. **d** Kaplan–Meier curve of mice survival (left) and tumour volume (right) after subcutaneous injection of E.G7-OVA cells (*n* = 7 independent animals; left, log-rank; right, two-way ANOVA). **e**, **f** Population (**e**) and representative flow cytometry plots (**f**) of tetramer^+^CD8^+^ T cells in splenocytes at the endpoint. Data in **e**, *n* = 4 independent animals, one-way ANOVA followed by Tukey’s multiple comparisons post hoc test, *p* < 0.0001. All data (**d**, right panel; **e**) are presented as mean + S.D.

**Fig. 6:**
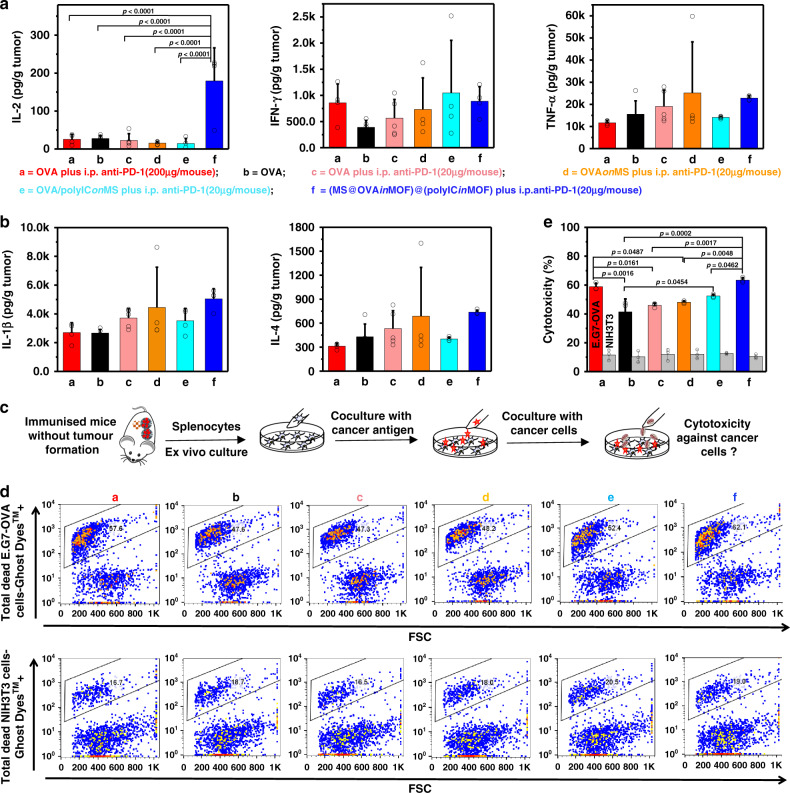
Cytokines secretion in tumour sites and OVA-specific cytotoxic CD8^+^ T-cell killing. **a, b** Cytokines in tumour at the endpoint (**a, d, e, f**, *n* = 4 independent animals; **b, c,** *n* = 5 independent animals; one-way ANOVA followed by Tukey’s multiple comparisons post hoc test; IL-2, *p* < 0.0001). **c** A schematic representation of antigen-specific cytotoxic T lymphocyte assay. The splenocytes were obtained from mice at the endpoint and cocultured with CFSE - stained live E.G7-OVA cancer cells or healthy NIH3T3 cells at the ratio of E/T = 10 and the specificity of cytotoxic CD8^+^ T cells against OVA were analysed using Ghost Dye™ Violet 450 staining and flow cytometry. **d** Representative flow cytometry plots of the splenocytes derived from different mice against E.G7-OVA cancer cells or healthy NIH3T3 cells. **e** Cytotoxicity of the splenocytes derived from different mice against E.G7-OVA cancer cells or healthy NIH3T3 cells (*n* = 3 independent samples, one-way ANOVA followed by Tukey’s multiple comparisons post hoc test; E.G7-OVA, *p* = 0.0002). All data (**a, b, e**) are presented as mean + S.D.

We explored combination immunotherapy using the subcutaneous (s.c.) administration of MOF-gated MS cancer vaccines plus intraperitoneal (i.p.) injection of anti-PD-1 Ab (Figs. 5 and 6a, b; Supplementary Figs. 31–33). A therapeutic mouse tumour model was established by s.c. injecting E.G7-OVA cancer cells into the right flank region of the mice (C57BL/6), followed by the s.c. injection of cancer vaccines into the left flank region plus i.p. injection of the anti-PD-1 Ab at a dose of 0, 20, or 200 μg per mouse on days 3, 7, 14, and 21 post-tumour inoculation. The pre-established tumours on the right flank were designated distant tumours without direct treatment since the subsequent cancer immunotherapy was administrated at different body sites. Mice were divided into nine treatment groups as shown in Table 1.

**Table 1:** Experimental parameters for combination immunotherapy of MOF-gated MS vaccination and PD-1 blockade (Figs. 5, 6 and S31–S34).

Group	Sample administered (s.c.)	Dose (/mouse, s.c.)	Dose (/mouse, i.p.)
a	Free OVA	OVA (100 μg)	Anti-PD-1 Ab (200 μg)
b	Free OVA	OVA (100 μg)	–
c	Free OVA	OVA (100 μg)	Anti-PD-1 Ab (20 μg)
d	OVA*on*MS	OVA, 100 μg; MS, 600 μg	Anti-PD-1 Ab (20 μg)
e	OVA/polyIC*on*MS	OVA, 100 μg; polyIC, 100 μg; MS, 600 μg	Anti-PD-1 Ab (20 μg)
f	(MS@OVA*in*MOF)@(polyIC*in*MOF)	OVA, 100 μg; polyIC, 100 μg; MS@MOF, 600 μg	Anti-PD-1 Ab (20 μg)
g	OVA*on*MS	OVA, 100 μg; MS, 600 μg	Anti-PD-1 Ab (200 μg)
h	OVA/polyIC*on*MS	OVA, 100 μg; polyIC, 100 μg; MS, 600 μg	Anti-PD-1 Ab (200 μg)
i	(MS@OVA*in*MOF)@(polyIC*in*MOF)	OVA, 100 μg; polyIC, 100 μg; MS@MOF, 600 μg	Anti-PD-1 Ab (200 μg)

Combination of MOF-gated MS vaccination with PD-1 blockade at a low dose significantly improves the therapeutic antitumour effect. Mice treated with (MS@OVA*in*MOF)@(polyIC*in*MOF) plus a low dose of i.p. anti-PD-1 (group f) show a higher survival rate and a smaller tumour volume than those treated with only free OVA (group b), free OVA plus a low dose of i.p. anti-PD-1 (group c), OVA*on*MS plus a low dose of i.p. anti-PD-1 (group d) and OVA/polyIC*on*MS plus a low dose of i.p. anti-PD-1 (group e). More importantly, mice treated with (MS@OVA*in*MOF)@(polyIC*in*MOF) plus a low dose of i.p. anti-PD-1 (group f) exhibit comparable tumour suppression to those treated with free OVA plus a conventional and 10 times higher dose of i.p. anti-PD-1 (group a). To understand the mechanisms underlying the significant antitumour therapeutic effects, we analysed the T-cell populations primed by the therapeutic vaccines (Fig. 5e, f). Mice treated with (MS@OVA*in*MOF)@(polyIC*in*MOF) plus a low dose of i.p. anti-PD-1 (group f) show significantly higher tetramer^+^CD8^+^ populations in splenocytes, than those treated with free OVA plus a high dose of anti-PD-1 Ab (group a), only free OVA (group b), free OVA plus a low dose of i.p. anti-PD-1 (group c), OVA*on*MS plus a low dose of i.p. anti-PD-1 (group d), and OVA/polyIC*on*MS plus a low dose of i.p. anti-PD-1 (group e). Administration of (MS@OVA*in*MOF)@(polyIC*in*MOF) plus a low dose of i.p. anti-PD-1 (group f) stimulates IL-2 secretion in the tumour of mice. Furthermore, MOF-gated MS vaccines comprising OVA and polyIC were used with a high dose of systemic anti-PD-1 Ab at 200 μg/mouse (Supplementary Figs. 32 and 33). Mice treated with (MS@OVA*in*MOF)@(polyIC*in*MOF) plus a high dose of i.p. anti-PD-1 (group i) show a higher survival rate and a smaller tumour volume, than those treated with free OVA plus a high dose of i.p. anti-PD-1 (group a), only free OVA (group b), OVA*on*MS plus a high dose of i.p. anti-PD-1 (group g), and OVA/polyIC*on*MS plus a high dose of i.p. anti-PD-1 (group h). Mice treated with (MS@OVA*in*MOF)@(polyIC*in*MOF) plus a high dose of i.p. anti-PD-1 (group i) show the highest tetramer^+^CD8^+^ populations in the spleen among all groups.

Overall, the MOF-gated MS as an adjuvant elicited antitumour immune responses more effectively than MS without MOF. An efficient cancer vaccine adjuvant should help antigen delivery to draining lymph nodes, enhance DC maturation and cross-presentation, and lead to robust CD8^+^ T-cell responses. Here, the MOF-gated MS effectively encapsulates the cancer antigen and immunopotentiator, prevents their off-target release, and enhances their targeted delivery to APCs and lymph nodes, resulting in the increase in tumour-specific CD8^+^ T populations, compared with the MS without MOF. We hypothesise that the pH-responsive gatekeeper property associated with MOF contributes to the efficient delivery of the high-molecular-weight antigen and immunopotentiator loading into MS having pore sizes as large as several tens nm.

### Specific cytotoxic T lymphocyte assay for E.G7-OVA cancer cells

To characterise the antigen-specific cell killing activity of CD8^+^ T cells against E.G7-OVA cells, splenocytes were collected from the mice at the endpoint and subcultured with IL-2 and OVA for 7 days in vitro (Fig. 6c–e, Supplementary Figs. 33–35). NIH3T3 fibroblasts and PC-12 pheochromocytoma cells were used as the controls. The splenocytes from mice treated with (MS@OVA*in*MOF)@(polyIC*in*MOF) plus a low dose of i.p. anti-PD-1 (group f) show significantly higher cytotoxicity against E.G7-OVA lymphoma cells expressing OVA than those from mice treated with groups free OVA plus a high dose of i.p. anti-PD-1 (group a), only free OVA (group b), free OVA plus a low dose of i.p. anti-PD-1 (group c), OVA*on*MS plus a low dose of i.p. anti-PD-1 (group d), and OVA/polyIC*on*MS plus a low dose of i.p. anti-PD-1 (group e). The splenocytes from mice treated with (MS@OVA*in*MOF)@(polyIC*in*MOF) plus a high dose of i.p. anti-PD-1 (group i) show the highest cytotoxicity against E.G7-OVA lymphoma among all groups. In contrast, splenocytes from all groups show a mild cytotoxicity against NIH3T3 fibroblasts and PC-12 pheochromocytoma cells without significant differences. CD8^+^ cytotoxic T lymphocytes (CTL) are the main effector cells in cell-mediated antitumour immunity^31,34^. CD8^+^ cytotoxic T lymphocytes in spleen derived from mice immunised with a (MS@OVAinMOF)@(polyICinMOF) cancer vaccine show higher specific killing ability against E.G7-OVA lymphoma cells expressing the epitope of OVA (SIINFEKL).

### Biocompatibility of MOF-gated MS

To confirm the safety profiles, healthy C57Bl/6J mice were subcutaneously administered with 1 mg of MS@MOF or MOF, and blood biochemical and tissue compatibility analyses were carried out (Supplementary Fig. 36). The saline-administrated group was used as the control. No obvious hepatic or renal toxicity is observed for MOF-gated MS and MOF as indicated by alanine aminotransferase (ALT) and aspartate aminotransferase (AST) levels for hepatic function and creatinine (CRE) and blood urea nitrogen (BUN) levels for renal function as compared with those for saline. Histological sections of the kidney, spleen, heart, liver, and lung derived from mice administrated with saline, MOF or MS@MOF exhibit no significant difference, suggesting no obvious tissue toxicity.

## Discussion

Cancer immunotherapies are increasingly recognised to be a promising strategy to elicit systemic immune responses and establish wide-spectrum treatment regimens for a variety of tumour types, since they aim to target the immune system rather than the tumour itself^13,35^. Combination immunotherapy based on synergetic effects between cancer vaccines and immune checkpoint blockade therapy can decrease the dose of immune checkpoint blockade as low as 1/10 while maintaining the effectiveness with minimising the possible immunotoxicity and therapeutic cost. The synergetic effects of suppressing tumour growth and activating antitumor immunity arise from enhancement of immunogenicity of cancer antigens and activation of antitumour CD8^+^ T-cell responses owing to the vaccine component, as well as remedy of immunosuppressive condition owing to the checkpoint blockade.

The integration of an antigen, immunopotentiator, and adjuvant into one particle with a pH switch for locally administered cancer vaccines is considered to enable the codelivery of these components to the same APCs, the colocalization and retention of loaded components in lymph nodes, highly efficient antigen cross-presentation, and the maximisation of cancer-antigen-specific T-cell response while preventing their entry into the systemic circulation, suppressing the initiation of undesirable stimulation in the blood or tissues, and improving the safety profiles^35,36^. The results in this study suggest that MOF-gated MS hold significantly higher capability to facilitate the intracellular uptake of cancer antigens by APCs, deliver the cancer antigens to lymph nodes, enhance antigen cross-presentation, promote the T-cell activation, and cytokine secretion, than MS nanoadjuvants. For MS nanoparticles with large pores and open channels, they provide the space to accommodate cancer antigens and immunopotentiators. However, when putting the molecules-loaded MS nanoparticles into the release buffer or injecting them into the body, the open porous structure of MS will result in the rapid leakage of the loaded components. Benefiting from the protective function of MOF as the gatekeeper, MOF-gated MS will greatly prevent the premature leakage of the encapsulated cancer antigens and immunopotentiators in the injection sites, since the MOF-gated MS exhibits small or negligible amounts of release at pH of ~7.4. MOF-gated MS showing a slow release of encapsulated cancer antigens and immunopotentiators in a neutral environment and a rapid release in an acidic environment will greatly promote the delivery of cancer antigens and immunopotentiators to pivotal APCs, the activation and trafficking of APCs to nearby tumour-draining lymph nodes, the presentation of digested fragments to naïve T cells, the clonal expansion of immune cells such as CD4^+^ and CD8^+^ T cells, the cytokine secretion to gain helper functions and thus the eradication of tumour cells.

The synergistic effects between cancer vaccines and checkpoint inhibitory antibodies occur to attack cancer cells and achieve the effective therapeutic activities against tumours. Systemic administration of anti-PD-1 Ab can alter the tumour microenvironment by blocking immunosuppressive signals. However, checkpoint blockade cancer therapy only exerts its effects when the tumours are immunogenic in patients, which might explain the low response rate of checkpoint blockade (~10–40%) in clinical trials. The prerequisite for the anti-PD-1 Ab to exert its effect is that the cancer antigen-specific anti-tumour immune response has been initiated. Thus, the external stimulation with cancer vaccines will be critical in strengthening the immunogenicity of tumor antigen, stimulating anti-tumour immunity, enhancing CD4^+^ and CD8^+^ T-cell populations and promoting Th1 cytokine secretion.

Vaccination using MOF-gated MS encapsulating OVA and polyIC greatly decreases the systemic dose of anti-PD-1 Ab when the vaccination and PD-1 blockage are combined. Under normal circumstances in a healthy body, the immune system can recognise cancer antigens and kill the cancer cells. However, once a tumour occurs in the body, tumour immunosuppressive microenvironments obstruct immune recognition and the process of cancer-immunity cycle^37^ owing to the weak immunogenicity of cancer antigens, creating negative regulatory pathways and other mechanisms. Vaccination using MOF-gated MS encapsulating OVA and polyIC efficiently triggers anti-tumour immune responses, and at the same time, the administration of anti-PD-1 Ab at a low dose blocks the immunosuppressive pathways. The synergistic effects of MOF-gated MS cancer vaccines and the anti-PD-1 checkpoint blockade make it easier to break down the immune equilibrium between promotive and suppressive factors, overcome the activation energy barrier associated with the immunosuppressive tumour microenvironment, and surmount the cancer-immune set point^38^. Then, the cancer-immunity cycle will be reinitiated, which covers a series of steps, including the capture of cancer antigens by APCs, antigen presentation to T cells, priming and activation of effector T cells, trafficking of effector T cells to tumours, infiltration of effector T cells into the tumour bed, recognition of cancer cells by T cells, killing of cancer cells, release of cancer antigens and so on^37^.

## Conclusions

In summary, cancer vaccines made from MOF-gated nanoadjuvants in combination with low-dose checkpoint blockade therapy are promising for cancer treatment. Inspired by the superior biomimetic mineralisation encapsulation capability of MOF for biomolecules and excellent intrinsic immune-shaping properties of MS, we fabricate MOF-gated nanoadjuvants to obtain the targeted delivery of immunology-associated large molecules to draining lymph nodes and navigate antitumour immunity. A combination of MOF-gated vaccine with systemic anti-PD-1 Ab administration successfully decreases the dose of anti-PD-1 Ab to 1/10 while maintaining the antitumour effectiveness. Notably, the MOF-gated MS delivery system is expected to be widely applicable for various therapeutic agents ranging from peptides, nucleic acids, molecule immunopotentiators, chemotherapeutic drugs to imaging contrast agents.

## Methods

### Physicochemical characterisation

The nanoadjuvants were observed using a field emission scanning electron microscope (FE-SEM, JEOL) after being coated with platinum and using transmission electron microscope (TEM, JEOL). The hydrodynamic diameter of nanoadjuvants was analysed by a dynamic light scattering photometer (DLS-8000HAL, Otsuka Electronics). The phases of nanoadjuvants were analysed using a powder X-ray diffractometer employing CuKα X-ray (Model RINT 2500, Rigaku). The zeta potential of nanoadjuvants was analysed using a Delta Nano C Particle Analyzer (Beckman Coulter Inc, America) by dispersing particles in calcium and magnesium - free phosphate - buffered saline (PBS(−)). The nitrogen gas (N_2_) adsorption-desorption isotherm of nanoadjuvants was measured by a surface area and porosity analyser (TriStar II, Micromeritics, America) and the BET specific surface areas and pore size distributions were calculated subsequently.

### Nanoadjuvants synthesis

MOF, MOF-gated MOF, MS and MOF-gated MS were synthesised in the preliminary experiment as follows. MOF was synthesised by mixing 80 μL of Zn(NO_3_)_2_·6H_2_O (Wako) solution (×1.0, 0.69 M), 400 μL of water and 800 μL of 2-methylimidazole (Wako) solution (×1.0, 3.13 M) with sonication for 20 min in ice. The obtained products were centrifuged, washed with ultra-pure water, and dispersed in solution or freeze-dried. MOF-gated MOF was synthesised by mixing 400 μL of MOF core suspension in aqueous solution at a concentration of 6 mg/mL, 80 μL of Zn(NO_3_)_2_·6H_2_O solution (×0.2, 0.138 M; ×0.4, 0.276 M; ×0.5, 0.345 M) and 800 μL of 2-methylimidazole solution (×0.2, 0.626 M; ×0.4, 1.252 M; ×0.5, 1.565 M) with sonication for 20 min in ice. The obtained products were centrifuged, washed with water, and dispersed in solution or freeze-dried.

MS were synthesised using a soft-templating method^39^. Typically, hexadecyltrimethylammonium p-toluenesulfonate (CTAT, Sigma-Aldrich) and triethanolamine (TEA, Sigma-Aldrich) were added into ultrapure water with stirring at 70 °C and tetraethoxysilane (TEOS, Wako, Japan) was slowly added. The molar ratio of the reaction mixture was 1.00 TEOS: 0.06 CTAT: 0.026 TEA: 80 H_2_O, respectively. The reaction mixture was continuously stirred for 2 h to obtain a precipitate. The obtained product was centrifuged, washed with ultrapure water/ethanol, dried and heat-treated at 550 °C for 5 h. MOF-gated MS was synthesised by mixing 400 μL of MS suspensions (0.033 M; 0.100 M; 0.166 M), 80 μL of Zn(NO_3_)_2_·6H_2_O solution (×0.1, 0.069 M; ×0.3, 0.207 M; ×0.4, 0.276 M; ×0.5, 0.345 M; ×0.6, 0.414 M) and 800 μL of 2-methylimidazole solution (×0.1, 0.313 M; ×0.3, 0.939 M; ×0.4, 1.252 M; ×0.5, 1.565 M; ×0.6, 1.878 M), respectively. Then, the products were centrifuged, washed with water, and dispersed in solution or freeze-dried.

### Encapsulation of model antigens and immunopotentiators into nanoadjuvants

In the preliminary experiments, Ovalbumin (OVA, Sigma-Aldrich) as a model antigen was encapsulated into MOF to prepare OVA*in*MOF by mixing 400 μL of OVA aqueous solution (1 mg/mL, 5 mg/mL and 25 mg/mL), 80 μL of Zn(NO_3_)_2_·6H_2_O solution (×1.0, 0.69 M) and 800 μL of 2-methylimidazole solution (×1.0, 3.13 M) with sonication for 20 min in ice followed by being centrifuged, washed with water and dispersed in solution or freeze-dried. In addition, polyinosinic - polycytidylic acid (polyIC, InvivoGen) as an immunopotentiator was encapsulated into MOF using 400 μL of polyIC aqueous solution (1 mg/mL, 5 mg/mL and 25 mg/mL) instead of the OVA aqueous solution.

In the subsequent experiments, MOF-gated MS with and without encapsulating biomolecules were synthesised as follows. Typically, MOF-gated MS with and without encapsulating OVA were synthesised by mixing 600 μL of MS aqueous suspensions with and without OVA, 80 μL of Zn(NO_3_)_2_·6H_2_O solution (×0.2, 0.138 M) and 600 μL of 2-methylimidazole solution (×0.2, 0.626 M) with sonication for 20 min in ice followed by being centrifuged, washed with water and dispersed in solution or freeze-dried. The samples were named MS@(OVA*in*MOF) and MS@MOF, respectively. Also, MS@(Ferritin*in*MOF) was prepared by the same method using ferritin instead of OVA. Then, (MS@OVA*in*MOF)@(polyIC*in*MOF), (MS@OVA*in*MOF)@(anti-CTLA4*in*MOF) and (MS@OVA*in*MOF)@MOF were prepared by mixing 600 μL of MS@(OVA*in*MOF) aqueous suspensions with and without polyIC or anti-CTLA4, 80 μL of Zn(NO_3_)_2_·6H_2_O solution (×0.2, 0.138 M) and 600 μL of 2-methylimidazole solution (×0.2, 0.626 M) with sonication for 20 min in ice followed by being centrifuged, washed with water, and dispersed in solution or freeze-dried.

### Quantitative approach of particles mass and biomolecules amounts

The mass of MS and MOF in MOF-gated MS is calculated by measuring average weight before and after synthesis reaction, with the exclusion of the dissolution of MS itself (*n* = 3). The mass of MOF is calculated by measuring average weight before and after synthesis reaction (*n* = 3). The concentrations of OVA, ferritin and anti-CTLA4 antibodies in solutions before and after loading are determined using a Bio-Rad protein assay kit (Bio-Rad Laboratories, Inc.). The concentrations of polyIC before and after loading are measured using an ultraviolet-visible spectrophotometer (V-550, JASCO). The encapsulation efficiencies of biomolecules (OVA, ferritin, polyIC and anti-CTLA4, etc.) are calculated by the following formula, respectively: biomolecule encapsulation efficiency = (Initial biomolecule concentration−biomolecule concentration after encapsulation)/Initial biomolecule concentration × 100%. The standard solutions of biomolecules, including OVA, ferritin, anti-CTLA4 antibodies and polyIC are obtained in the same concentration of 2-methylimidazole solution with synthesis parameter.

### Degradation of nanoadjuvants associated with release of biomolecules

MOF-gated MS or MOF with and without encapsulating OVA were synthesised. The final mass ratios of MS:MOF in MS@MOF and MS:MOF:OVA in MS@(OVA*in*MOF) are about 2.4:0.6 and 2.4: 0.6: 1, respectively. The final mass ratios of MOF:OVA in OVA*in*MOF are about 3: 1. In contrast, OVA*on*MS was prepared by mixing OVA solution and MS particles with a MS:OVA mass ratio of 3:1. Degradation of MS, MS@MOF, MOF, OVA*on*MS, MS@(OVA*in*MOF) and OVA*in*MOF samples contained in a bag of dialysis membrane in an acetate buffer (pH = 5) or a Tris-HCl buffer (pH = 7.4) at a particles-to-buffer ratio of 1 mg/mL was quantitatively analysed after incubation at 37 °C by measuring Si and Zn using an inductively coupled plasma atomic emission spectrometer (ICP-AES: SPS7800, Seiko Instruments). The OVA release was determined in an acetate buffer (pH = 5) or a Tris-HCl buffer (pH = 7.4) at a particles-to-buffer ratio of 3 mg/mL at 37 °C.

The MS@(polyIC*in*MOF) and polyIC*in*MOF samples were synthesised by the method same as that for the above MS@(OVA*in*MOF) and OVA*in*MOF samples except that the mass ratios of MS:MOF:polyIC and MOF:polyIC are 2.4:0.6:0.5 and 3:0.5, respectively. The polyIC*on*MS was prepared by mixing polyIC solution and MS particles with a MS:polyIC mass ratio of about 3:0.5. The polyIC release was determined in an acetate buffer or a Tris-HCl buffer at a particles-to-buffer ratio of 1 mg/mL at 37 °C.

In addition, Tris-HCl buffer supplemented with 10% serum was used as a third type of media to test the degradation of nanoadjuvants associated with release of molecules using same protocol as those for an acetate buffer or a Tris-HCl buffer. In the serum solution, ferritin was used as a model biomolecule, due to the overlap of OVA and serum in spectra. The release of ferritin was quantitatively analysed by measuring Fe using ICP-AES. The release of polyIC in serum solution was tested using a StrandBriteTM Green Fluorimetric RNA Quantitation Kit (AAT Bioquest).

The standard solutions of biomolecules for release experiments, including OVA, ferritin and polyIC, are obtained in acetate buffer, Tris-HCl buffer or Tris-HCl buffer supplemented with 10% serum, according to the corresponding experimental parameters.

### Sodium dodecyl sulfate-polyacrylamide gel electrophoresis

OVA-loaded nanoadjuvants, including OVA*on*MS, MS@(OVA*in*MOF) and OVA*in*MOF, were dispersed in PBS(−) with the final concentration of 200 ng/μL OVA and 800 ng/μL particles, respectively. Free OVA was used as control. To prepare the supernatant samples, OVA-loaded nanoadjuvants were suspended in PBS(−) for 1 h and centrifuged at 13,000 rpm for 10 min. Then, OVA-loaded nanoadjuvants or supernatant samples were mixed with 2× SDS–PAGE sample buffer, incubated at 50 °C for 10 min, loaded into the gel and subjected to electrophoresis at 30 mA for 70 min running with 1× Tris-Glycine-SDS buffer according to the manufacturer’s instructions. The gels were visualised by staining with Rapid Stain Coomassie Brilliant Blue kit.

### Cellular uptake and activation of dendritic cells in vitro

Bone marrow derived dendritic cells (BMDCs) were obtained from mice femurs^40^. After removing red blood cells, I-A/I-E and phycoerythrin-conjugated anti-CD4, CD8 expressing cells, the left cells were cultured in RPMI 1640 (Gibco) containing 10% fetal bovine serum and 20 ng/mL granulocyte macrophage colony-stimulating factor (GM-CSF, Bioreagent). The BMDCs were collected on day 9. In all, 2 × 10^5^ BMDCs were precultured in glass bottom dish or 96-well plate for 6 h. Nanoadjuvants prepared using OVA or fluorescein-conjugated OVA (fOVA, Life technologies), where they were OVA*on*MS, MS@(OVA*in*MOF), OVA*in*MOF, fOVA*on*MS, MS@(fOVA*in*MOF) and fOVA*in*MOF, were added to the BMDCs culture media at a particle concentration of 30 μg/mL and a OVA or fOVA concentration of 5 μg/mL. After overnight culture, the BMDCs were stained with Hoechst (Thermo Fisher) for cell nuclei and observed by a confocal laser microscope (Leica). The quantitative analysis of cellular uptake fluorescence images was calculated using image J software. TNF-α and IL-1β in the supernatant were quantified using mouse ELISA kit (BD Biosciences) according to the manufacturer’s instructions. To further measure activation of BMDCs, 2 × 10^6^ BMDCs were cocultured in 24-well plate with free OVA, OVA*on*MS, MS@(OVA*in*MOF) and OVA*in*MOF at a particle concentration of 30 μg/mL and a OVA concentration of 5 μg/mL, respectively. After 3 days’ culture, the BMDCs were collected using Trypsin-EDTA, blocked with anti-CD16/CD32 Ab (2.4G2, BioLegend) with 1/100 dilution and stained with anti-mouse OVA257-264 (SIINFEKL) peptide bound to H-2Kb Ab, anti-mouse MHC II (I-A/I-E) Ab, anti-mouse CD80 Ab, anti-mouse CD40 Ab and anti-mouse CD197(CCR7) Ab (BioLegend) with 1/50 dilution. Flow cytometry was carried out using FACSAria (BD Bioscience, USA). For all flow cytometry experiments, 1–3 million cells per sample were collected for staining. Among them, at least 10–50 thousand cells per sample were used for flow cytometry analysis. Flowjo software was used to analyse the flow cytometry data.

In addition, to compare the difference between adsorption and encapsulation of fOVA or OVA, fOVA- or OVA- adsorbing MOF (MOF-ad) and fOVA- or OVA- encapsulating MOF by coprecipitation (MOF-en) were prepared, and added to the BMDCs culture at a particle concentration of 25 μg/mL and a OVA or F-OVA concentration of 5 μg/mL.

### Antigen retention at the injection sites and particle distribution in vivo

Alex Fluor 647-conjugated OVA (A647-OVA, Molecular Probes) or MS@(A647-OVA*in*MOF) was injected into the flank of the female C57Bl/6J mice (*n* = 3, every group; CLEA Inc.) at a A647-OVA dose of 100 μg/mouse and particle dose of 600 μg/mouse in 100 μL saline. At 6 h, 1 day and 3 day, the mice were observed using IVIS imaging system with excitation wavelength of 580 nm and emission wavelength of 680 nm. To clearly see the body distribution of nanoparticles, the main organs (nearby draining lymph node, spleen, lung, heart, kidney and liver) of mice were collected and observed using IVIS imaging system at day 1. Living image software was used to analyse the data. Moreover, ICP-AES measurement was used to quantitatively analyse the targeting distribution of nanoparticles (Si and Zn content) in nearby draining lymph node.

### APCs-mediated delivery to lymph nodes and cross-presentation of OVA in vivo

Female C57Bl/6J mice (3 mice, every group; CLEA Inc.) were immunised by injecting fOVA, fOVA*on*MS, MS@(fOVA*in*MOF) and fOVA*in*MOF subcutaneously into the left flank at particles and fOVA doses of 600 μg/mouse and 100 μg/mouse, respectively. The immunised mice were killed 16 h later, and the nearby draining lymph node was collected. The cryostat sections of lymph nodes were prepared, stained with DAPI and observed using a fluorescence microscope (Olympus BX51) with a highly-sensitive camera (Olympus DP74). Fluorescent images were acquired under identical parameter settings. The quantitative analysis of fluorescent images was calculated using image J software. Moreover, the draining lymph node was collected, milled, vortexed and filtered through a 40-μm cell strainer to obtain single-cell suspension. The single-cell suspension was washed with PBS(−) containing 0.5% bovine serum albumin (BSA). Non-specific staining was prevented by blocking the cells with anti-CD16/CD32 Ab (2.4G2, BioLegend) with 1/100 dilution. The cells were stained for 30 min with anti-mouse CD11c Ab and anti-mouse OVA257-264 (SIINFEKL) peptide bound to H-2Kb Ab (BioLegend) with 1/50 dilution. Flow cytometry was performed using FACSAria (BD Bioscience, USA).

### Prophylactic cancer immunotherapy

Twenty eight female C57Bl/6J mice (7 mice/group; 6 weeks old, CLEA Inc.) were divided into the following four groups: 1# saline group; 2# free OVA-anti-CTLA4 group (OVA, 100 μg/mouse; anti-CTLA4 Ab, Bio X Cell, 20 μg/mouse); 3# (OVA*in*MOF)@(anit-CTLA4*in*MOF) group (OVA, 100 μg/mouse; anti-CTLA4 Ab, 20 μg/mouse; MOF, 600 μg/mouse); and 4# (MS@OVA*in*MOF)@(anti-CTLA4*in*MOF) (OVA, 100 μg/mouse; anti-CTLA4 Ab, 20 μg/mouse; MS@MOF, 600 μg/mouse). Each individual subject in 100 μL saline was administered subcutaneously into the left flank of mice at day 0, 3 and 10. At day 14, the mice were challenged by live E.G7-OVA cells (5 × 10^5^ cells mouse^−1^) subcutaneously into the right flank. Tumour growth on the right flank of mice was monitored three times per week and continued for several weeks. Survival rate was statistically calculated based on tumour size <15 mm. The tumour volume was calculated by 1/2 × longest dimension × perpendicular dimension^2^.

### Combination cancer immunotherapy

First, live E.G7-OVA cells (2 × 10^5^ cells mouse^−1^) were injected subcutaneously into the right flank of sixty three female C57BL/6 mice (7 mice/group; 6 weeks old, CLEA Inc.). On day 3, 7, 14, 21 post-tumour inoculation, mice were divided into the following nine groups and injected with the following subjects in 100 μL saline: free OVA (100 μg/mouse) plus a high dose of i.p. anti-PD-1 Ab (Bio X Cell, 200 μg/mouse) in group a; only free OVA (100 μg/mouse) in group b; free OVA (100 μg/mouse) plus a low dose of i.p. anti-PD-1 Ab (20 μg/mouse) in group c; OVA absorbed on MS (OVA*on*MS: OVA, 100 μg/mouse; MS, 600 μg/mouse) plus a low dose of i.p. anti-PD-1 Ab (20 μg/mouse) in group d; OVA/polyIC adsorbed on MS (OVA/polyIC*on*MS: OVA, 100 μg/mouse; polyIC, 100 μg/mouse; MS, 600 μg/mouse) plus a low dose of i.p. anti-PD-1 Ab (20 μg/mouse) in group e; (MS@OVA*in*MOF)@(polyIC*in*MOF) (OVA, 100 μg/mouse; polyIC, 100 μg/mouse; MS@MOF particles, 600 μg/mouse) plus a low dose of i.p. anti-PD-1 Ab (20 μg/mouse) in group f, OVA*on*MS (OVA, 100 μg/mouse; MS, 600 μg/mouse) plus a high dose of i.p. anti-PD-1 Ab (200 μg/mouse) in group g; OVA/polyIC*on*MS (OVA, 100 μg/mouse; polyIC, 100 μg/mouse; MS, 600 μg/mouse) plus a high dose of i.p. anti-PD-1 Ab (200 μg/mouse) in group h; (MS@OVA*in*MOF)@(polyIC*in*MOF) (OVA, 100 μg/mouse; polyIC, 100 μg/mouse; and MS@MOF particles, 600 μg/mouse) into the left flank plus a high dose of i.p. anti-PD-1 Ab (200 μg/mouse) in group i. Tumour growth on the right flank of mice was monitored for several weeks. Survival rate was calculated based on tumour size <15 mm. The tumour volume was calculated by 1/2 × longest dimension × perpendicular dimension^2^.

### Cytokine contents in tumour sites and spleen

At the endpoint of prophylactic and combination cancer immunotherapy, the tumour sites and spleen were excised and lysed with a T-PER tissue protein extraction reagent (Thermo Fisher Scientific), and the amounts of cytokines in tumour sites were quantified using mouse ELISA kit (BD Biosciences) according to the manufacturer’s instructions.

### Analysis of antigen-specific T-cell populations

At the endpoint of prophylactic and combination cancer immunotherapy, splenocytes were collected from the spleen, milled, vortexed and filtered through a 40-μm cell strainer to obtain single-cell suspension. Anti-CD16/CD32 antibody (2.4G2, Biolegend) with 1/100 dilution was used to prevent the nonspecific staining. Anti-mouse CD8α Ab (BioLegend) and anti-mouse T-Select H-2Kb OVA Tetramer-SIINFEKL Ab (MBL) with 1/50 dilution were used to stain the cells for 30 min. Then, the intracellular cytokine was stained by anti-mouse IFN-γ Ab (BioLegend) with 1/50 dilution. Flow cytometry was performed for the cell suspensions using a FACSAria cell cytometer (BD Biosciences).

### Specific cytotoxic T lymphocyte assay for E.G7-OVA cancer cells

At the endpoint of combination cancer immunotherapy, mice from all groups were killed to harvest splenocytes. After 7 days of splenocytes subculture with 40 ng/mL mouse IL-2 and 20 μg/mL OVA, the splenocytes were cocultured with 5-(and -6)-Carboxyfluorescein diacetate succinimidyl ester (CFSE, Dojindo) - stained live E.G7-OVA cancer cells and NIH3T3 fibroblasts at effector cells/target cells ratio of 10, respectively. In addition, mouse from group f were cocultured CFSE - stained live E.G7-OVA cancer cells and PC-12 cancer cells at effector cells/target cells ratio of 0, 5, 10 and 20, respectively. The cells were then stained with Ghost Dye™ Violet 450 (Bay bioscience) 24 h later. The cytotoxicity of splenocytes against E.G7-OVA cancer cells, NIH3T3 fibroblasts and PC-12 cancer cells were analysed using a FACSAria cell cytometer (BD Biosciences), respectively. The cytotoxicity is calculated by the following formula: cytotoxicity = (total dead target cells−spontaneous dead target cell)/(total target cells−spontaneous dead target cell) × 100%.

### Biocompatibility of nanoadjuvants

To examine the in vivo safety, the saline, MOF-gated MS and MS (1 mg/mouse in 100 μL saline) were subcutaneously injected into the left flank of C57/BL6J mice (5 mice, every group; CLEA Inc.). Mice were euthanized 3 days later, and blood was harvested for blood haematology analysis. The organs, including kidney, spleen, heart, liver and lung, were collected, fixed in 10% neutral buffered formalin solution (Wako), embedded in paraffin and stained with hematoxylin and eosin.

### Statistics and reproducibility

The statistical significance of differences was calculated by log-rank test, Student’s *t* test or ANOVA with Tukey’s multiple comparisons *post hoc* test. A *p* value of <0.05 was considered statistically significant. Each experiment was repeated independently at least twice with similar results.

### Ethical issue

The animal experiments were permitted by the Ethical Committee of the National Institute of Advanced Industrial Science and Technology (AIST), Japan. All the animal experiments and feeding were carried out in accordance with the guidelines of the Ethical Committee of AIST, Japan.

### Reporting summary

Further information on research design is available in the Nature Research Reporting Summary linked to this article.

## Supplementary information

Supplementary Information

Reporting Summary

## Data Availability

Source data for Figs. 2, 3, 4, 5 and 6 and Supplementary Figs. 20, 22, 23, 25, 26, 27, 28, 30, 32, 33, 35 and 36 are provided as a Source Data file. All other relevant data are available in the article, Supplementary information, or from the corresponding author upon reasonable request. Source data are provided with this paper.

## Source data

Source Data
