# Supplementary material for: A nanoscale metal organic frameworks-based vaccine synergises with PD-1 blockade to potentiate anti-tumour immunity
Source: Nat Commun. 2020 Jul 31;11:3858. doi: 10.1038/s41467-020-17637-z (PMC7395732; doi:10.1038/s41467-020-17637-z)
Supplement: Supplementary file 1 — Supplementary Information [file 41467_2020_17637_MOESM1_ESM.pdf]

Supplementary information.

A nanoscale metal organic frameworks-based vaccine synergises with PD-1 blockade to potentiate anti-tumour immunity

Li et al.

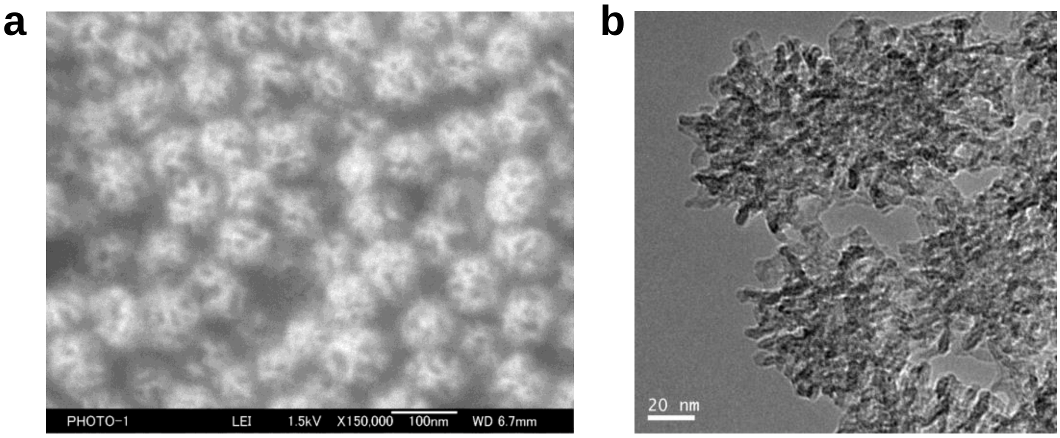

Supplementary Figure 1. (a, b) SEM (a) and (b) TEM images of MS.

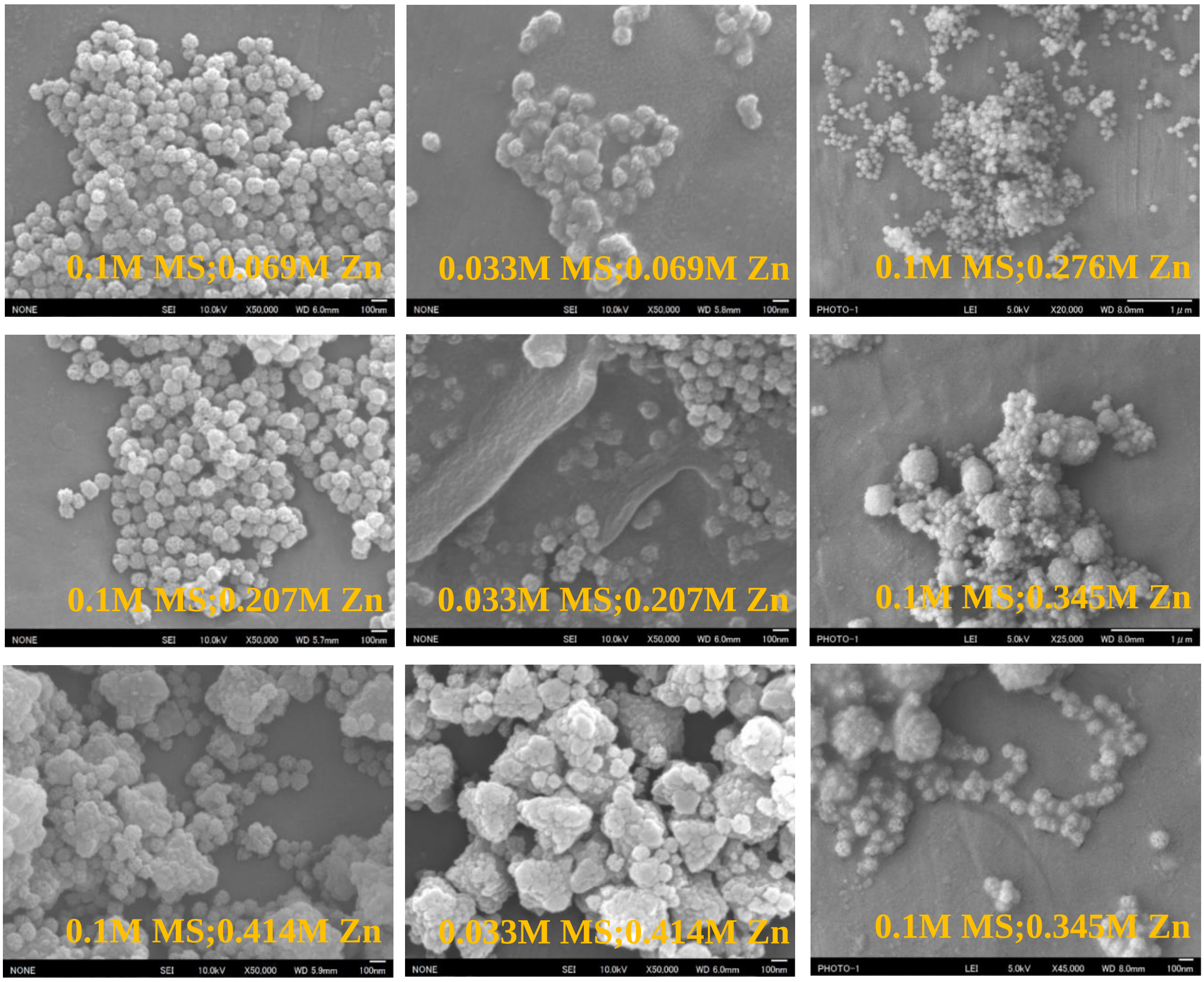

Supplementary Figure 2. SEM images of MS@MOF with different initial concentrations. In the synthesis process, MOF-gated MS was synthesized by mixing 400 μL of stellated fibrous MS suspensions (0.033M; 0.100M) and 80 μL of $Zn(NO_3)_2 \cdot 6H_2O$ solution (×0.1, 0.069M; ×0.3, 0.207M; ×0.4, 0.276M; ×0.5, 0.345M; ×0.6, 0.414M) followed by addition of 800 μL of 2-methylimidazole solution (×0.1, 0.313M; ×0.3, 0.939M; ×0.4, 1.252M; ×0.5, 1.565M; ×0.6, 1.878M), respectively.

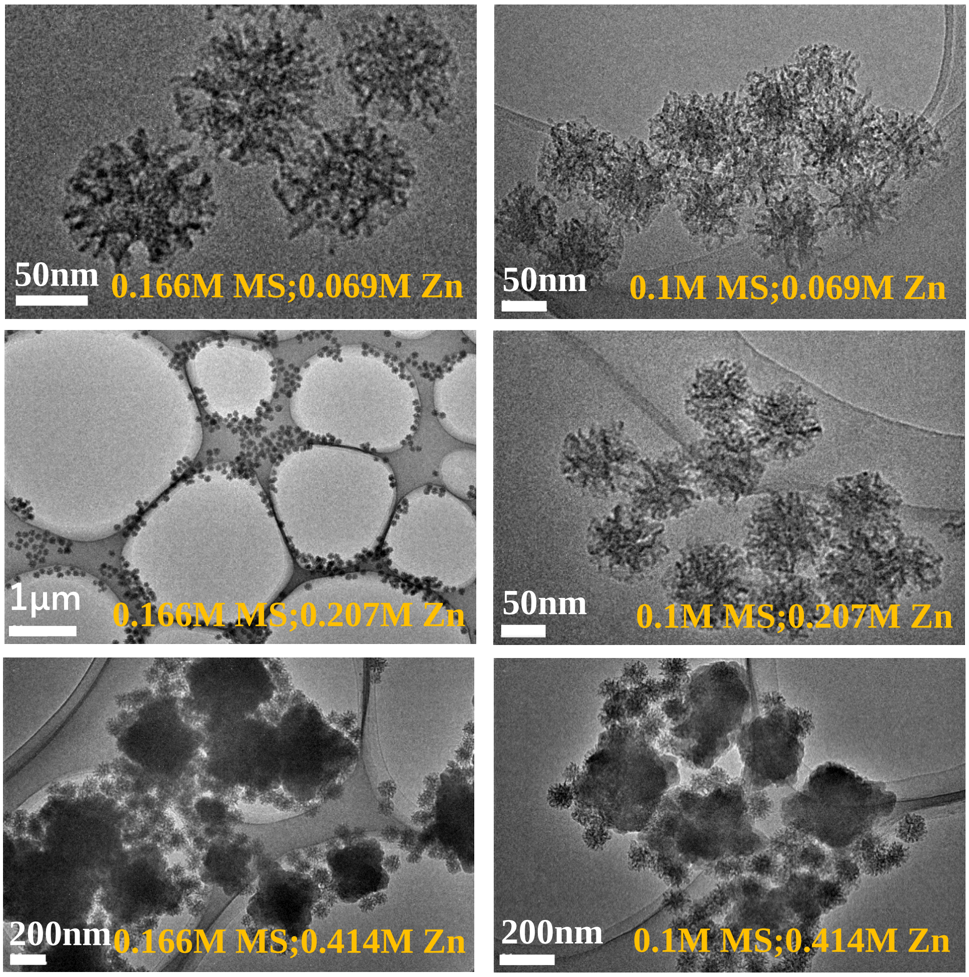

Supplementary Figure 3. TEM images of MS@MOF with different concentrations. In the synthesis process, MOF-gated MS was synthesized by mixing 400 μL of stellated fibrous MS suspensions (0.100M; 0.166M), 80 μL of $Zn(NO_3)_2·6H_2O$ solution (×0.1, 0.069M; ×0.3, 0.207M; ×0.6, 0.414M) and 800 μL of 2-methylimidazole solution (×0.1, 0.313M; ×0.3, 0.939M; ×0.6, 1.878M), respectively.

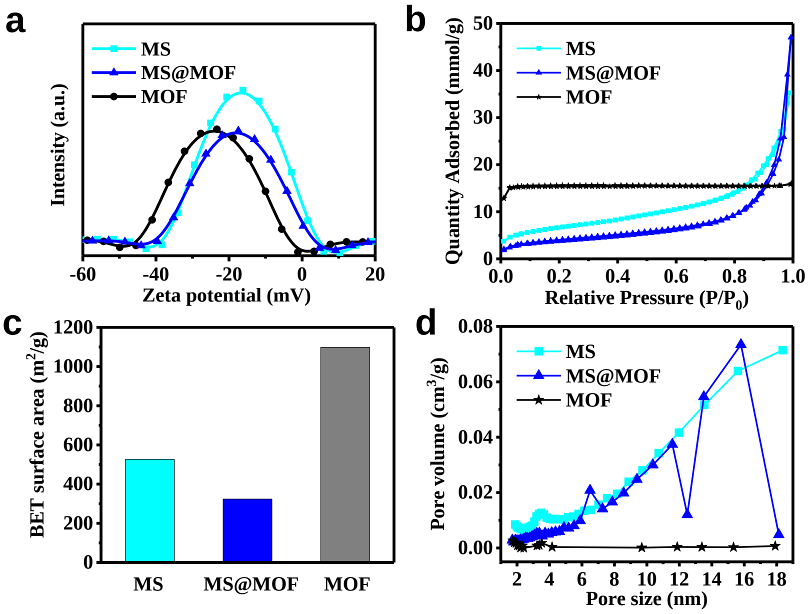

Supplementary Figure 4. (a) Zeta potentials of MS, MOF-gated MS and MOF. **(b-d)** N_2 adsorption-desorption isotherms (b), BET surface area (c) and pore size distribution (d) of MS, MS@MOF and MOF.

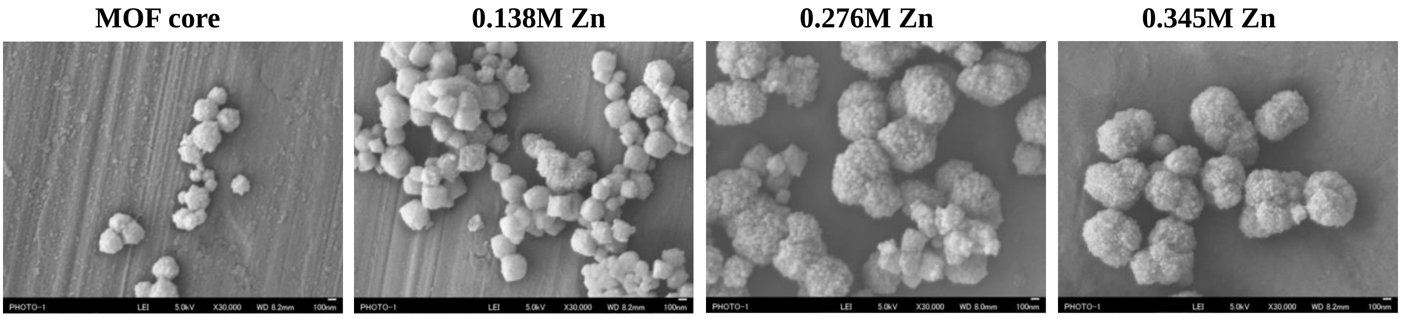

Supplementary Figure 5. SEM images of MOF and MOF@MOF.

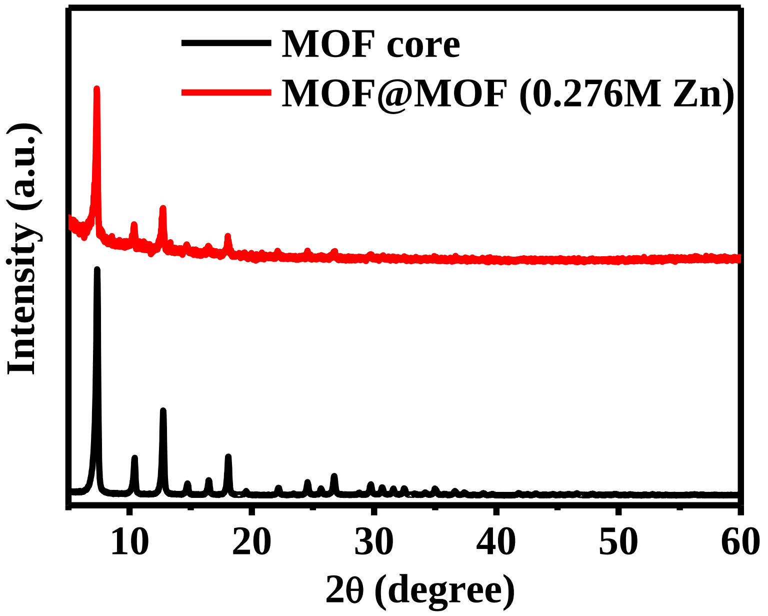

Supplementary Figure 6. XRD patterns of MOF and MOF@MOF.

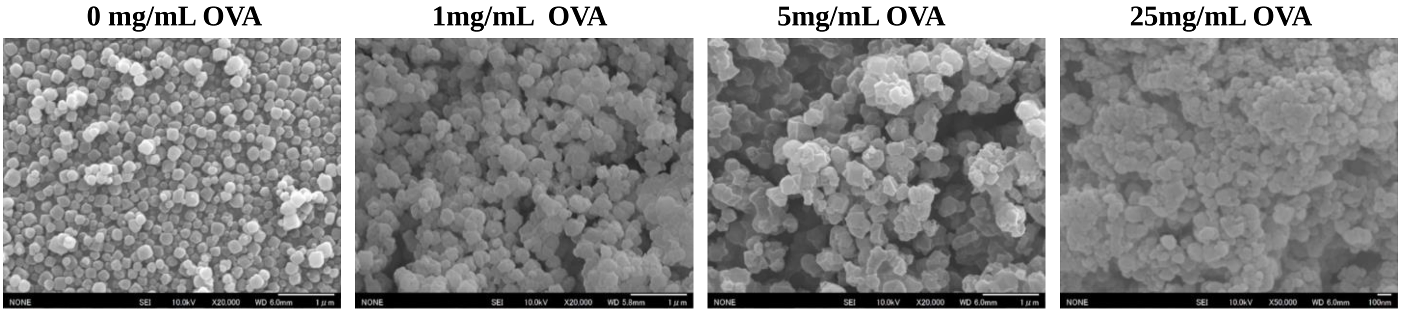

Supplementary Figure 7. SEM images of OVA*in*MOF with different OVA concentrations. In the synthesis process, OVA as a model antigen was encapsulated into MOF to prepare OVA*in*MOF by mixing 400 μL of OVA aqueous solution (1mg/mL, 5mg/mL, 25mg/mL), 80 μL of $Zn(NO_3)_2 \cdot 6H_2O$ solution (0.69M) and 800 μL of 2-methylimidazole solution (3.13M) with sonication for 20min in ice followed by being centrifuged, washed with water and freeze-dried.

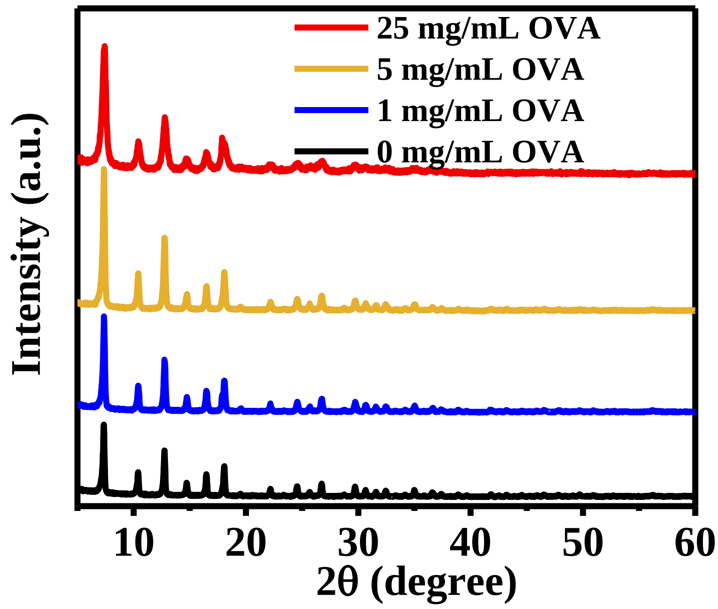

Supplementary Figure 8. XRD patterns of OVA*in*MOF with different OVA concentrations.

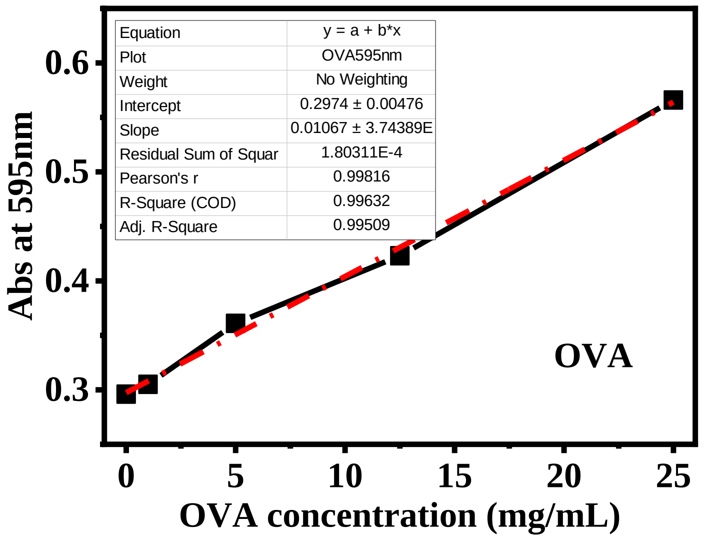

Supplementary Figure 9. Standard curves of OVA in 2-methylimidazole solution using a Bio-Rad protein assay kit. In detail, OVA primary solution (0, 1, 5, 12.5, 25mg/mL) were mixed with other reagents at the same ratio with the synthesis process except the use of water instead of $Zn(NO_3)_2 \cdot 6H_2O$ solution and then diluted 10 times with water. Thus, the absorbance at 595nm was tested after mixing samples and 5× diluted dye reagent at the volume ratio of 1:40 according to the manufacturer's protocal.

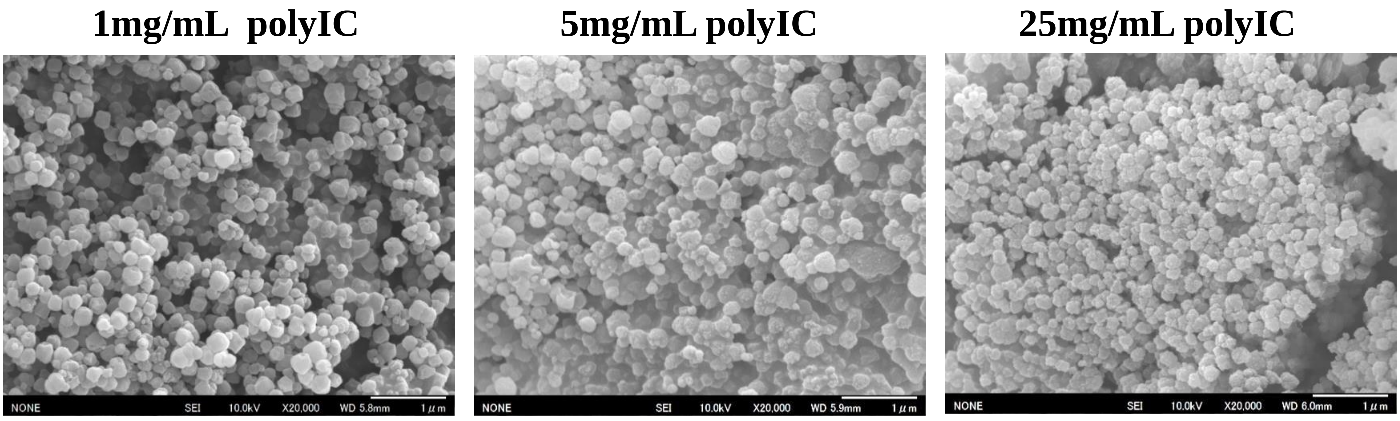

Supplementary Figure 10. SEM images of polyIC*in*MOF with different polyIC concentrations. In the synthesis process, polyIC as an immunopotentiator was encapsulated into MOF to prepare polyIC*in*MOF by mixing 400 μL of polyIC aqueous solution (1, 5, 25mg/mL), 80 μL of $Zn(NO_3)_2 \cdot 6H_2O$ solution (0.69M) and 800 μL of 2-methylimidazole solution (3.13M) with sonication for 20min in ice followed by being centrifuged, washed with water and freeze-dried.

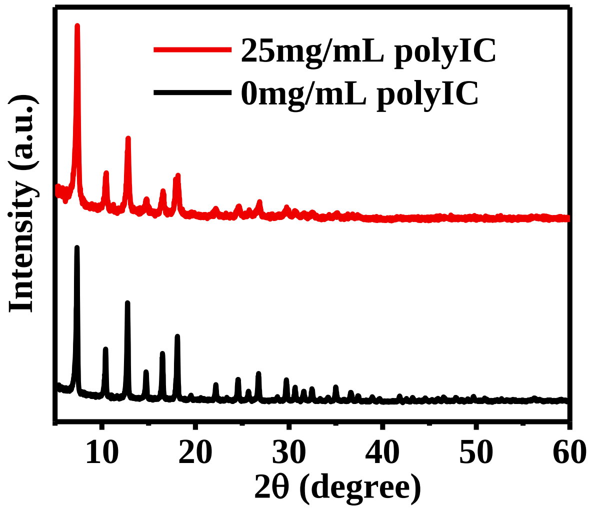

Supplementary Figure 11. XRD patterns of polyIC*in*MOF with different polyIC concentrations.

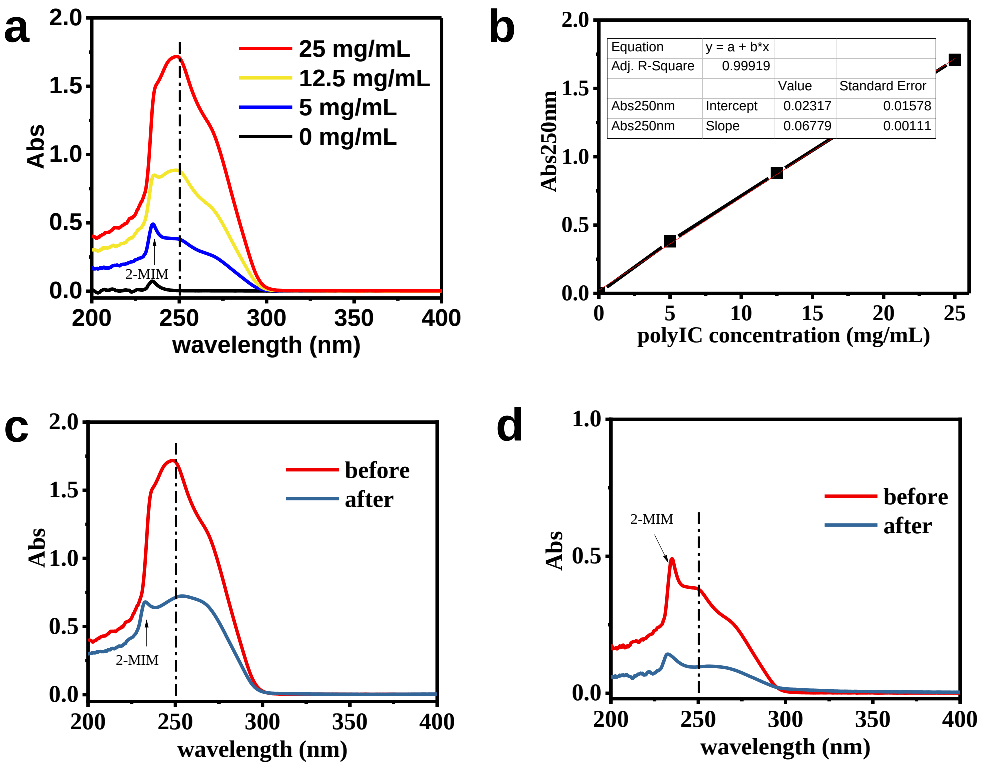

Supplementary Figure 12. (a, b) Standard UV-vis spectra of polyIC in 2-methylimidazole solution. In detail, polyIC solution (0, 5, 12.5, 25mg/mL) were mixed with other reagents at the same ratio with the synthesis process except the use of water instead of $Zn(NO_3)_2 \cdot 6H_2O$ solution and then diluted 200 times with water. **(c, d)** UV-vis spectra of synthesis solution before and after encapsulation when 25 mg/mL (c) and 5 mg/mL (d) polyIC solutions were used.

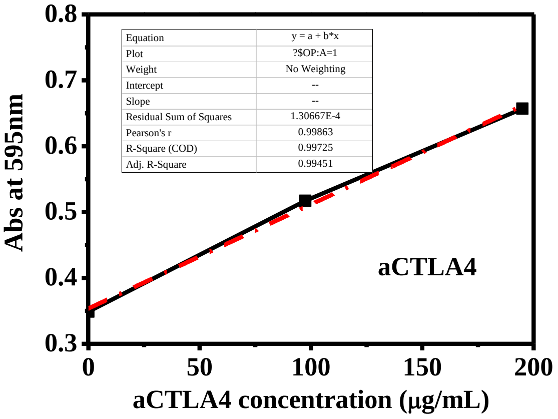

Supplementary Figure 13. Standard curves of anti-CTLA4 in 2-methylimidazole solution using a Bio-Rad protein assay kit. In detail, samples were mixed with 5× diluted dye reagent at the volume ratio of 1:20 and the absorbance at 595nm were measured according to the manufacturer’s protocal.

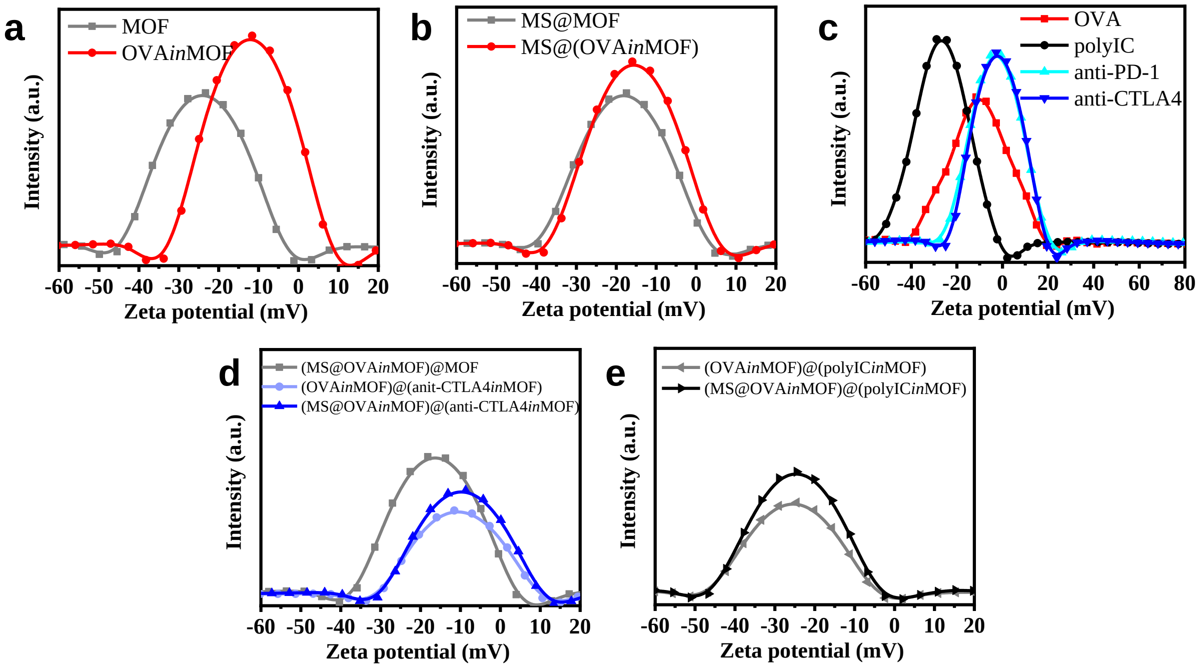

Supporting information. Fig S14. Zeta potentials of samples.

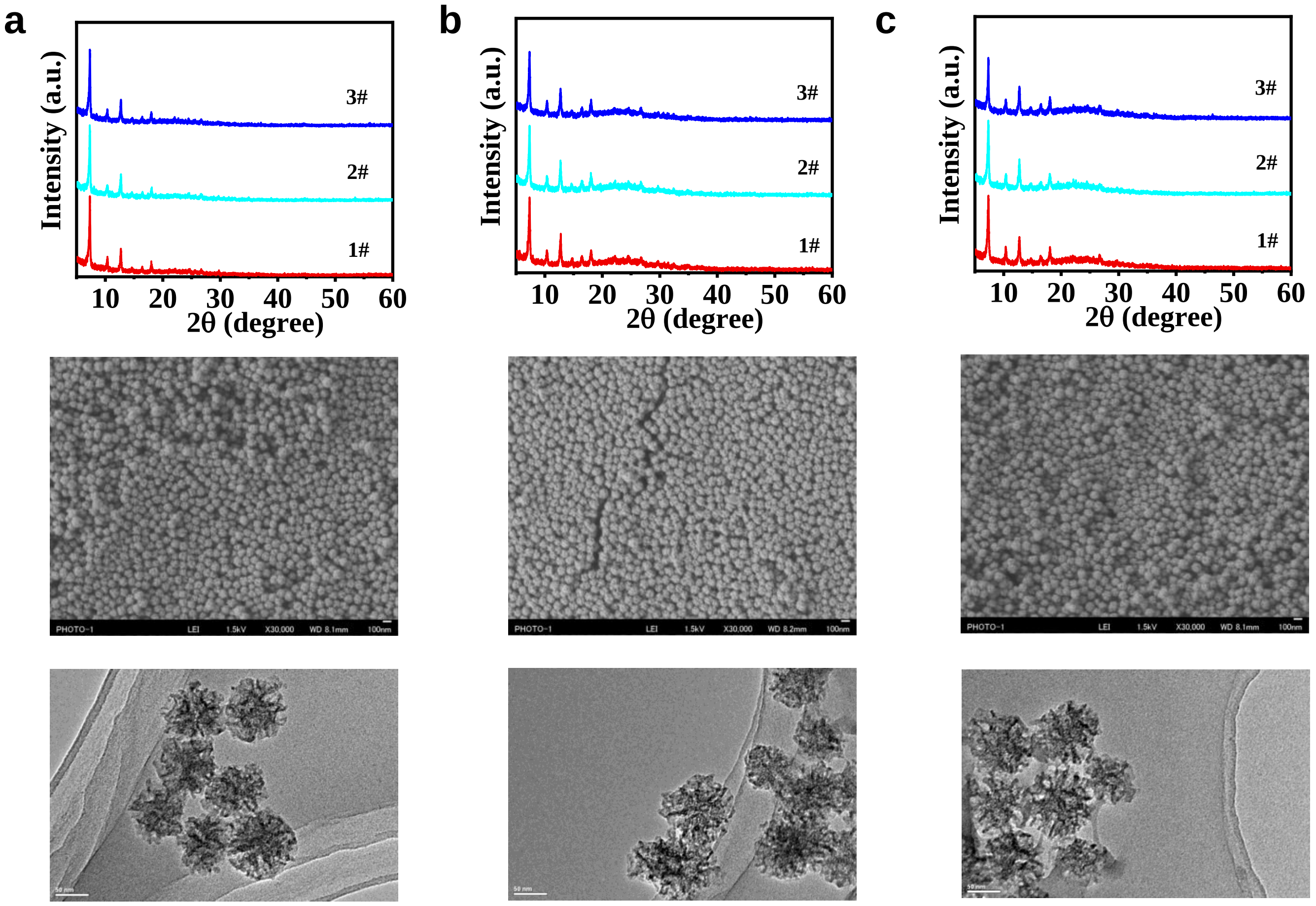

Supplementary Figure 15. (a-c) XRD patterns (1#, 2# and 3#: same samples from different batches), SEM images and TEM images of MS@(OVA*in*MOF) (a), (MS@OVA*in*MOF)@(polyIC*in*MOF) (b) and (MS@OVA*in*MOF)@(anti-CTLA4*in*MOF) (c).

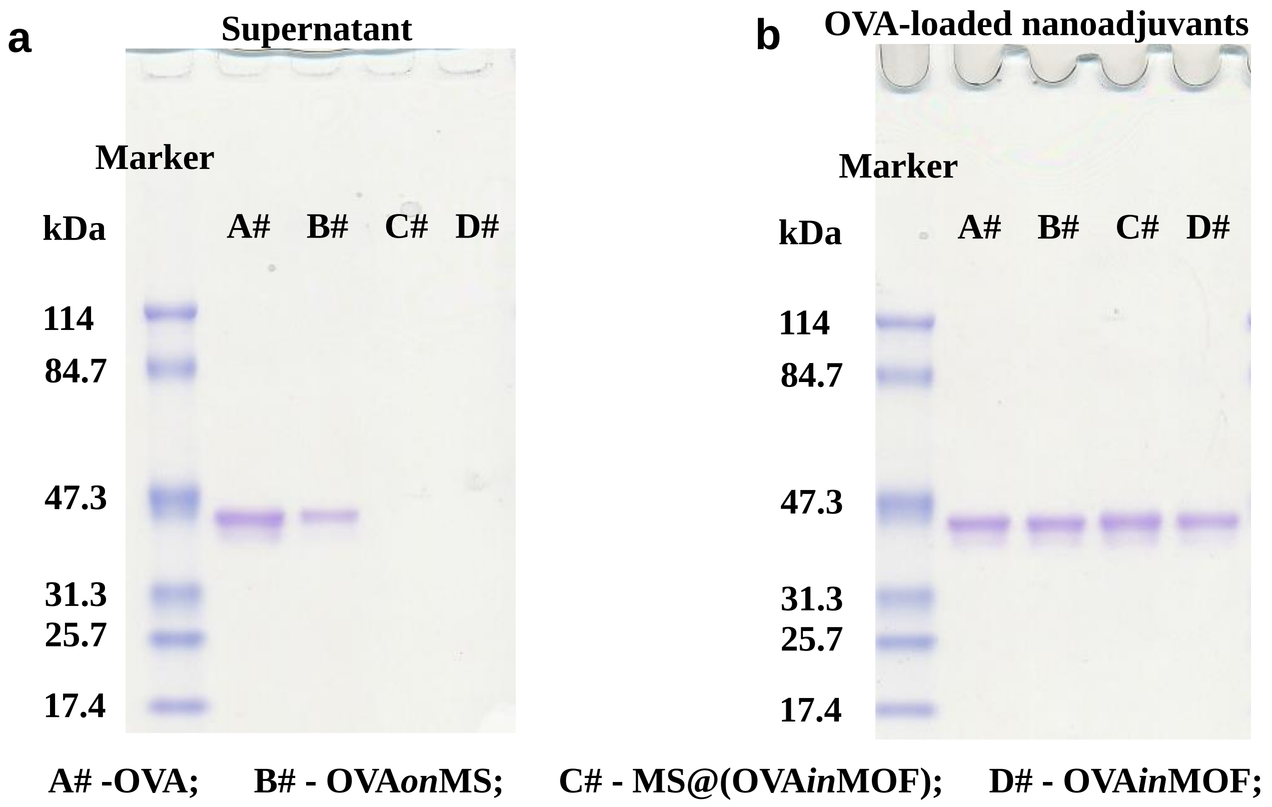

Supplementary Figure 16. SDS-PAGE gel electrophoresis of the supernatants collected after dispersing different samples in water for 1 h (a) and free OVA or OVA-loaded nanoadjuvants (b).

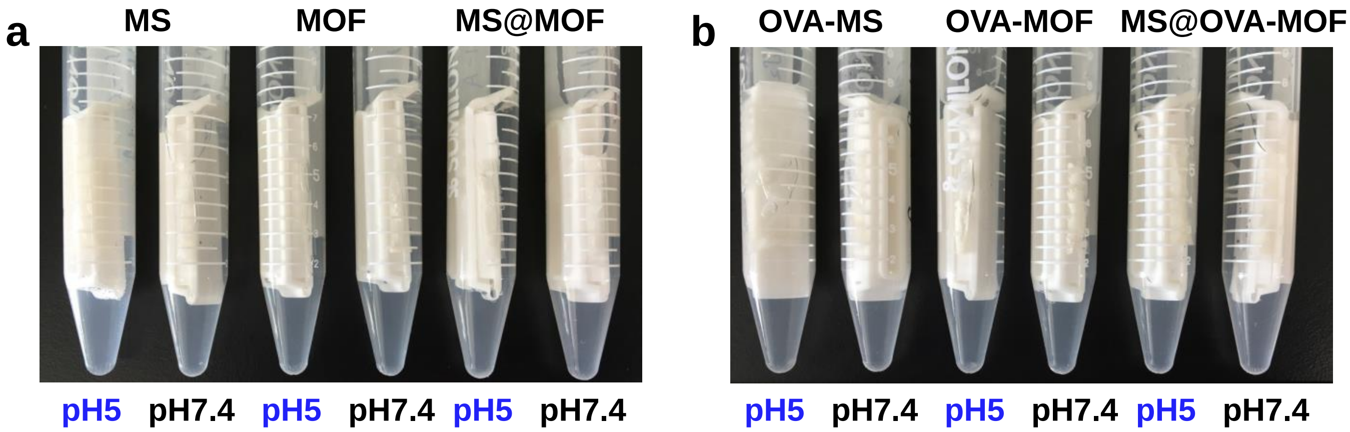

Supplementary Figure 17. (a, b) Degradation of different samples.

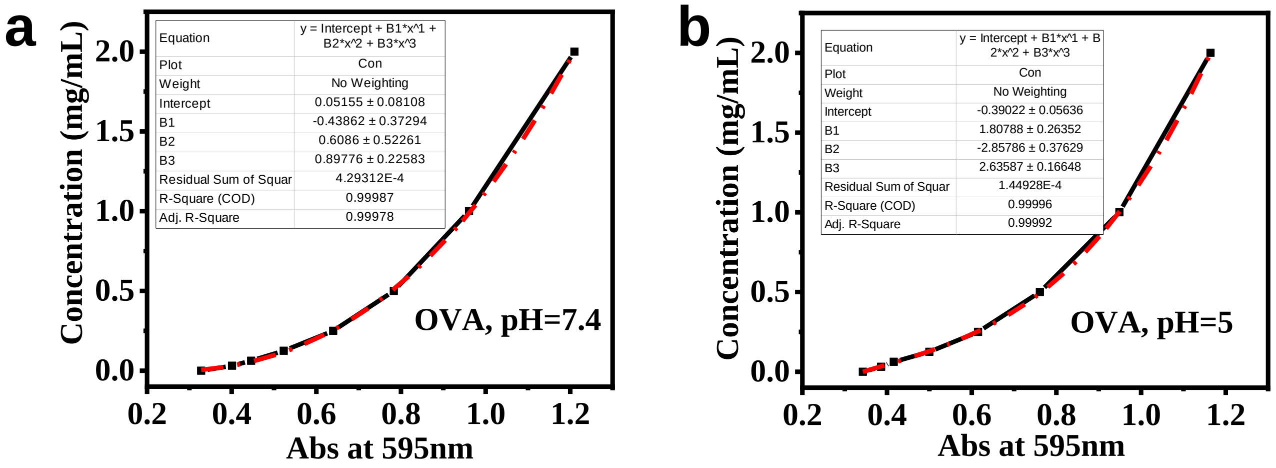

Supplementary Figure 18. (a, b) Standard curves of OVA in pH=5 (a) and pH=7.4 (b) buffer using a Bio-Rad protein assay kit. In detail, OVA solution in different buffer were mixed with diluted dye reagent at the volume ratio of 1:10 and the absorbance at 595nm were measured according to the manufacturer’s protocal.

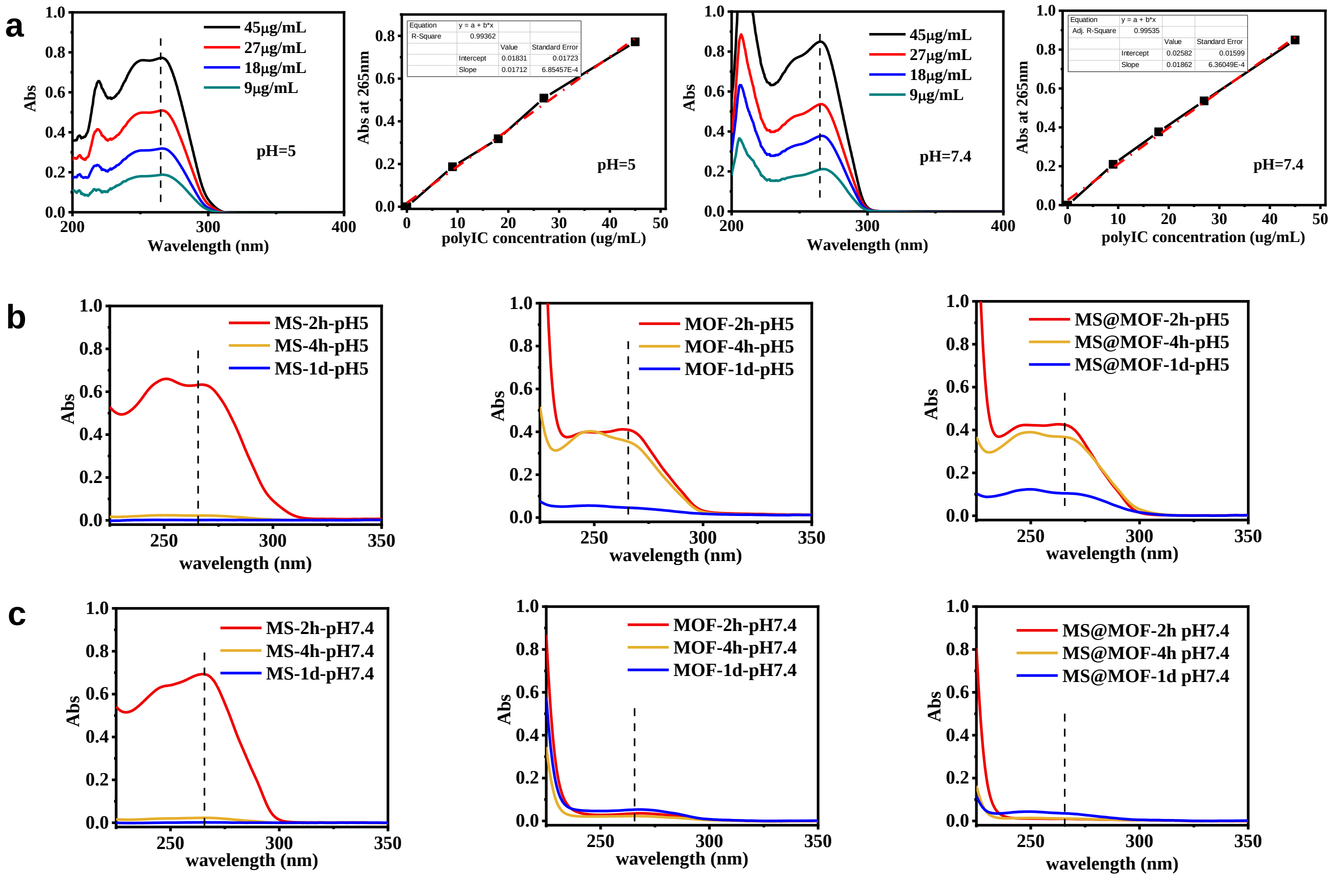

Supplementary Figure 19. (a-c) Standard curves (a) and release of polyIC in pH=5 (b) and pH7.4 (c) buffer using UV-vis spectroscopy.

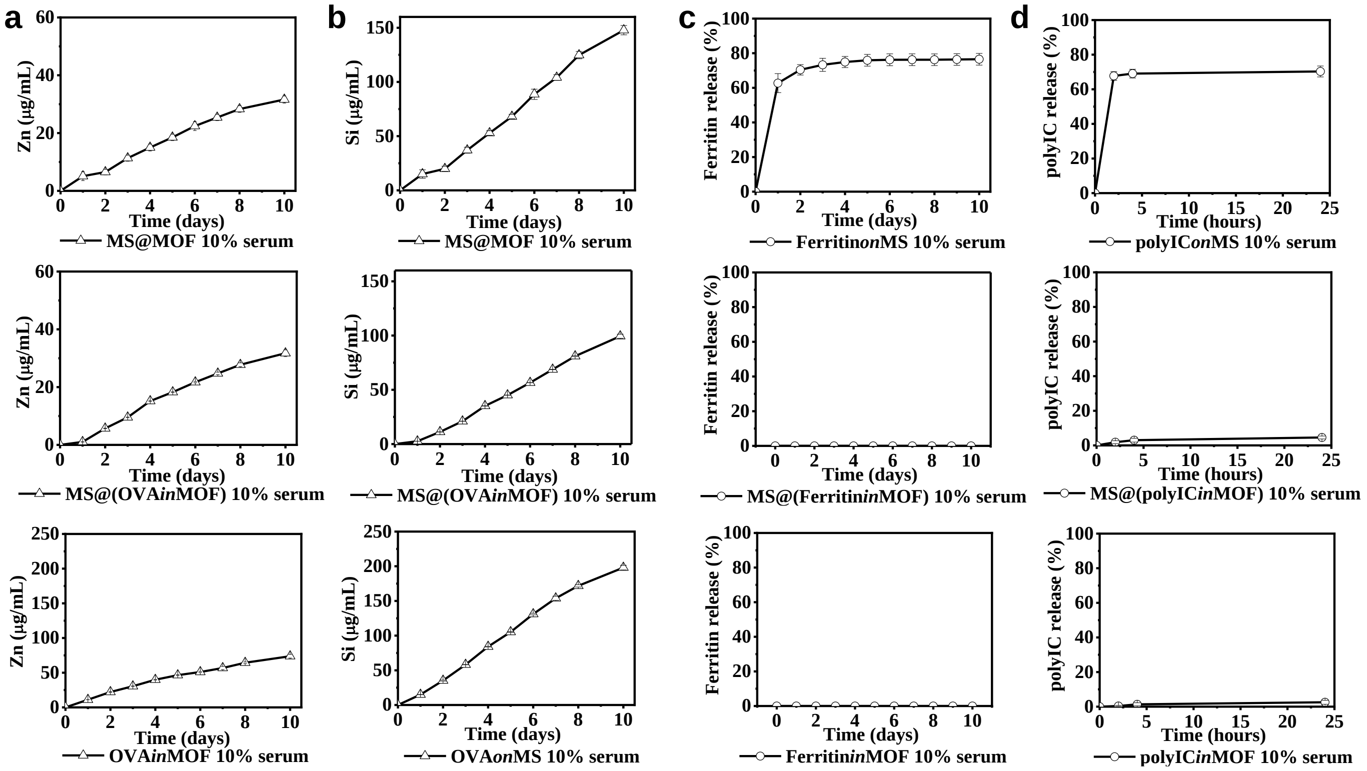

Supplementary Figure 20. (a-d) Degradation (a, b, n=3 independent samples) and biomolecule release curves (c, n=3; d, n=4 independent samples) of nanoadjuvants in Tris-HCl buffer (pH.7.4) supplemented with 10% serum. Data are presented as mean ± S.D.

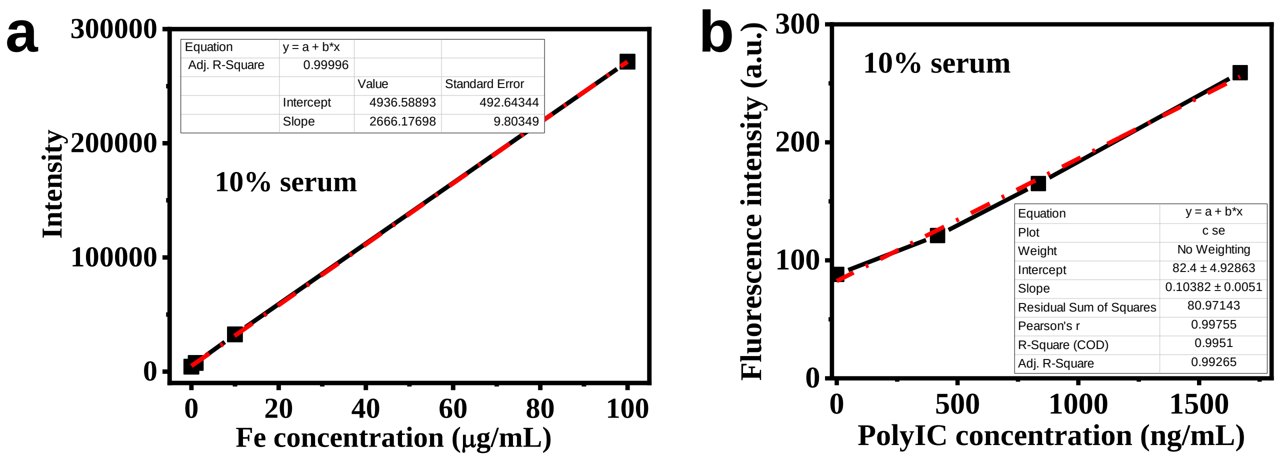

Supplementary Figure 21. (a) Standard curve of ferritin using ICP. **(b)** Standard curve of polyIC using StrandBriteTM Green Fluorimetric RNA Quantitation Kit in Tris-HCl buffer (pH.7.4) supplemented with 10% serum.

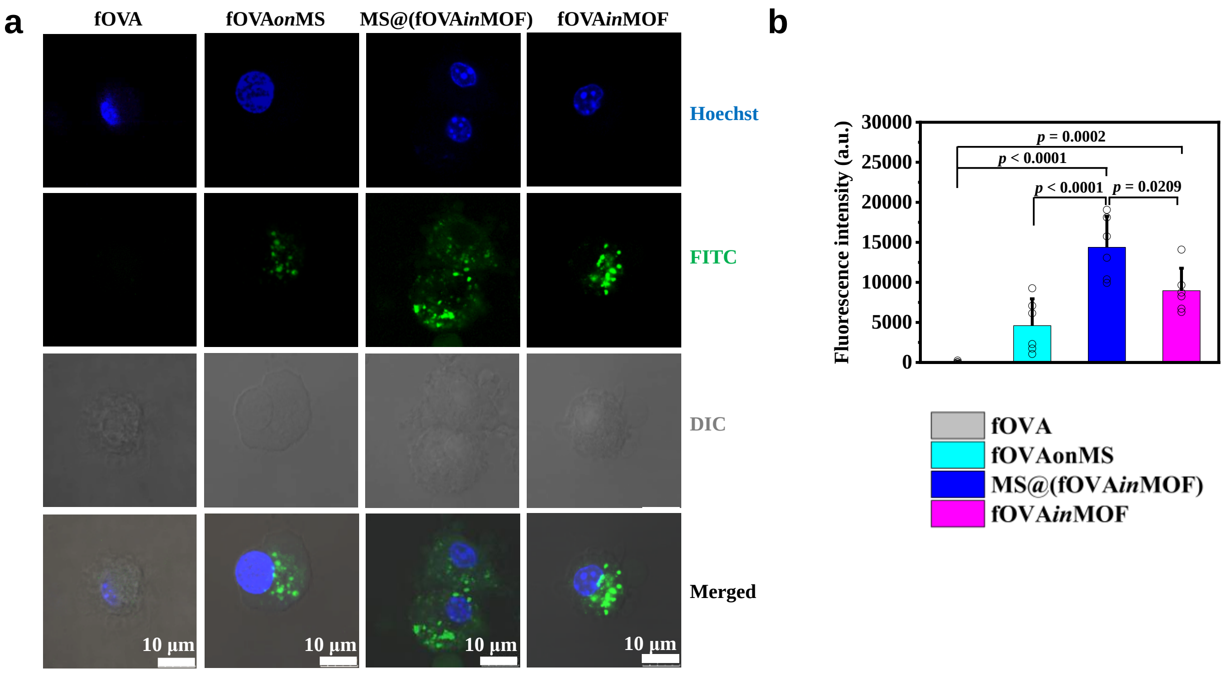

Supplementary Figure 22. (a, b) Confocal laser scanning microscopic images (a) and quantitative analysis (b, n=6 independent samples, one-way ANOVA followed by Tukey's multiple comparisons *post hoc* test, p<0.0001) of the cellular uptake of free fOVA, fOVA*on*MS, MS@(fOVA*in*MOF) and fOVA*in*MOF by BMDCs. Hoechst, blue; FITC-OVA (model cancer antigen), green. Data are presented as mean + S.D.

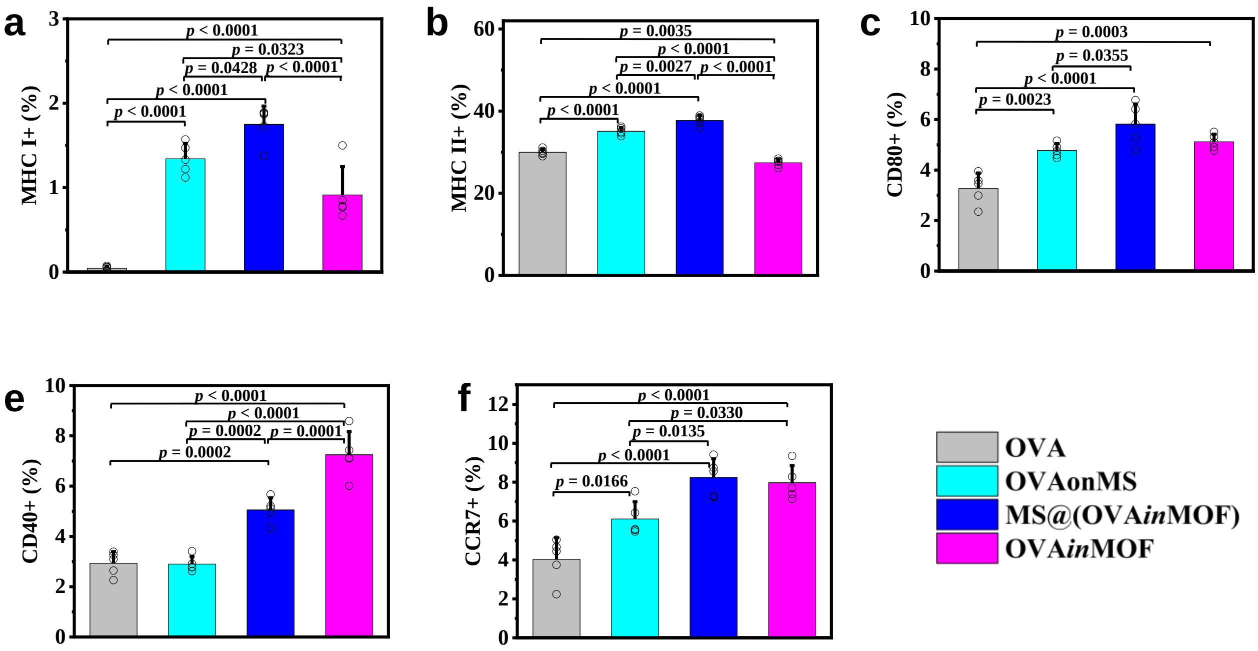

Supplementary Figure 23. (a-f) Quantitative analysis of BMDCs activation markers when culturing with different nanoadjuvants for 3 days (n=5 independent samples, one-way ANOVA followed by Tukey's multiple comparisons *post hoc* test; a, p<0.0001; b, p<0.0001; c, p<0.0001; d, p<0.0001; e, p<0.0001). Data are presented as mean + S.D.

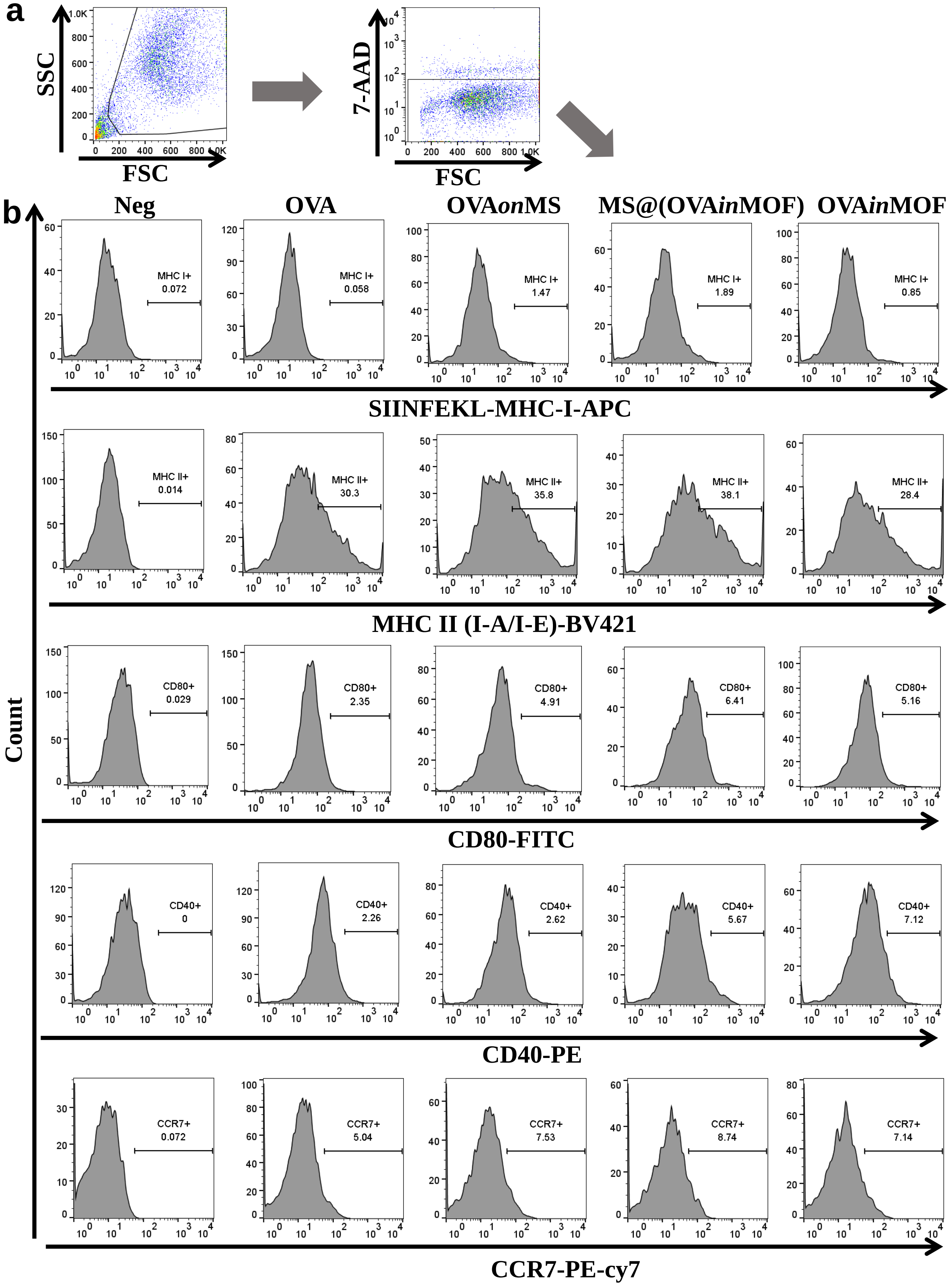

Supplementary Figure 24. (a) Gating hierarchy. **(b)** Representative flow cytometry histogram of control and BMDCs activation marker when culturing with different nanoadjuvants for 3 days.

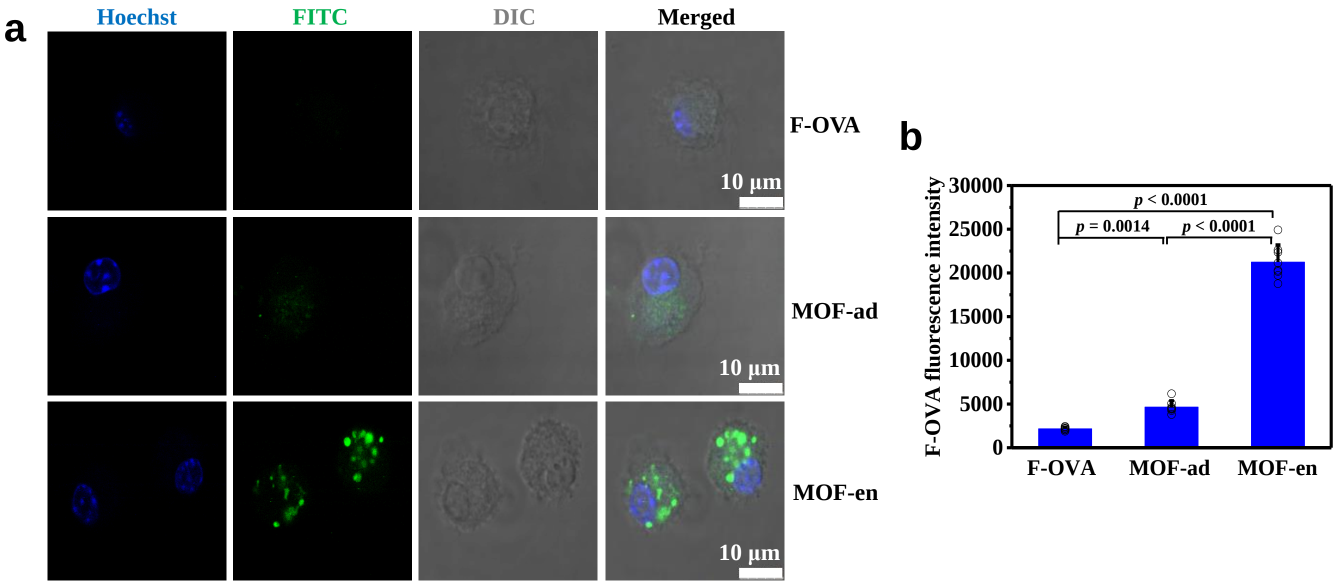

Supplementary Figure 25. (a) Confocal images of cellular uptake by BMDCs: free F-OVA; F-OVA-adsorbing MOF (MOF-ad); F-OVA-encapsulating MOF (MOF-en). **(b)** Quantitative analysis of F-OVA fluorescent intensity for F-OVA, MOF-ad and MOF-en (n=8 independent samples, one-way ANOVA followed by Tukey's multiple comparisons *post hoc* test, p<0.0001). Data are presented as mean + S.D.

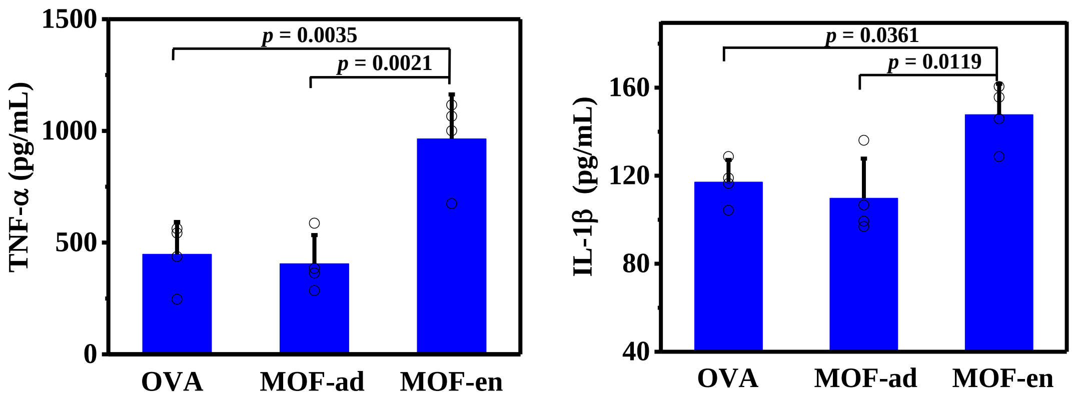

Supplementary Figure 26. Cytokine secreted from BMDCs when exposed to free OVA, OVA-adsorbing MOF (MOF-ad) and OVA-encapsulating MOF (MOF-en) (n=4 independent samples, one-way ANOVA followed by Tukey's multiple comparisons *post hoc* test; TNF-α, p=0.0013; IL-1β, p=0.0109). Data are presented as mean + S.D.

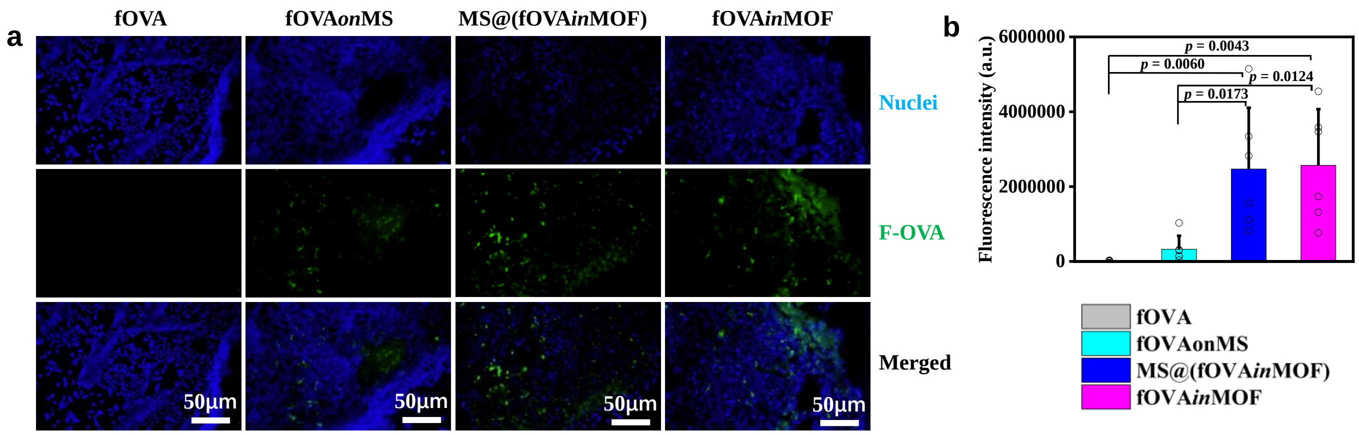

Supplementary Figure 27. (a, b) Representative cryosection images of lymph nodes (a) and quantitative analysis of F-OVA fluorescence intensity (b, n=6 independent samples, one-way ANOVA followed by Tukey's multiple comparisons *post hoc* test, p=0.0006) in mice vaccinated with: fOVA, fOVA*on*MS, MS@(fOVA*in*MOF) and fOVA*in*MOF. DAPI, blue; F-OVA, green. Data are presented as mean + S.D.

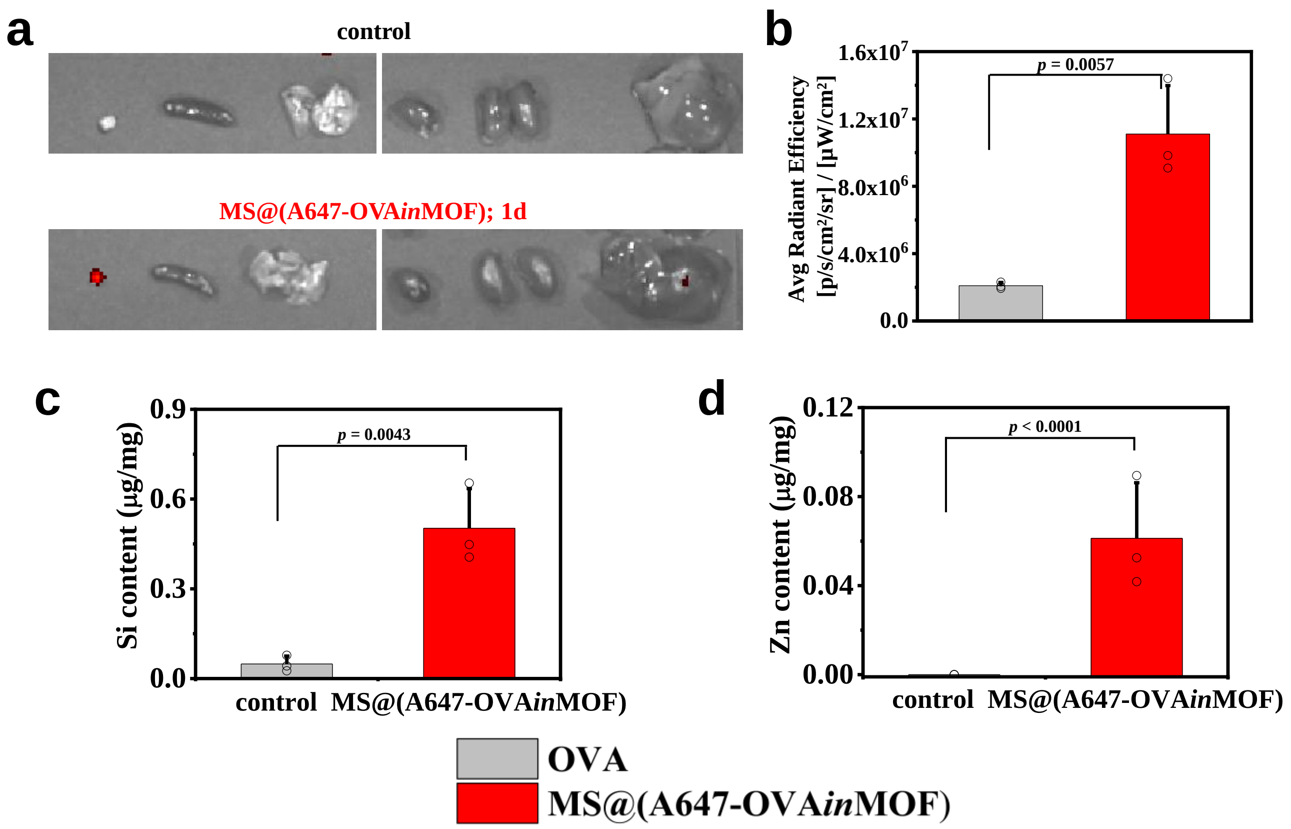

Supplementary Figure 28. (a, b) Representative images of MS@(A647-OVA*in*MOF) distribution in body observed by IVIS fluorescent imaging system (a) and quantitative analysis of MS@(A647-OVA*in*MOF) accumulation in nearby draining lymph node (b, n=3 independent animals; Student's t-test, two-tailed) after 1d injection. **(c, d)** MS@(A647-OVA*in*MOF) distribution in nearby draining lymph node tested by ICP analysis (n=3 independent animals; Student's t-test, two-tailed) after 1d injection. Data are presented as mean + S.D.

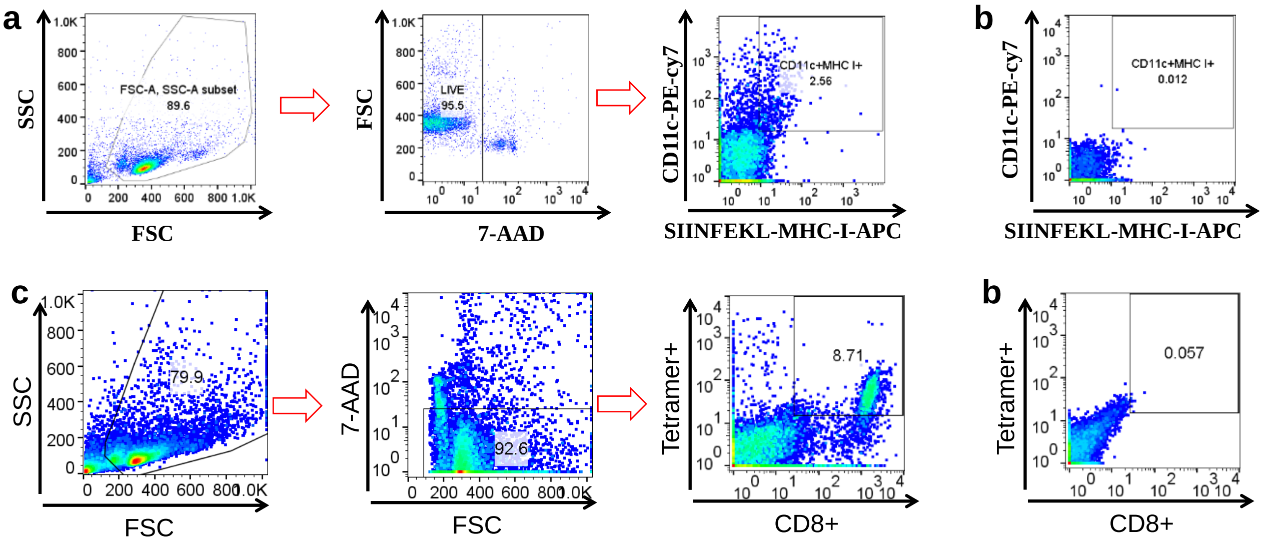

Supplementary Figure 29. (a, b) Gating hierarchy (a) and control dot plots (b) of Fig. 3h. **(c, d)** Gating hierarchy (c) and control dot plots (d) of Fig. 4 d-f.

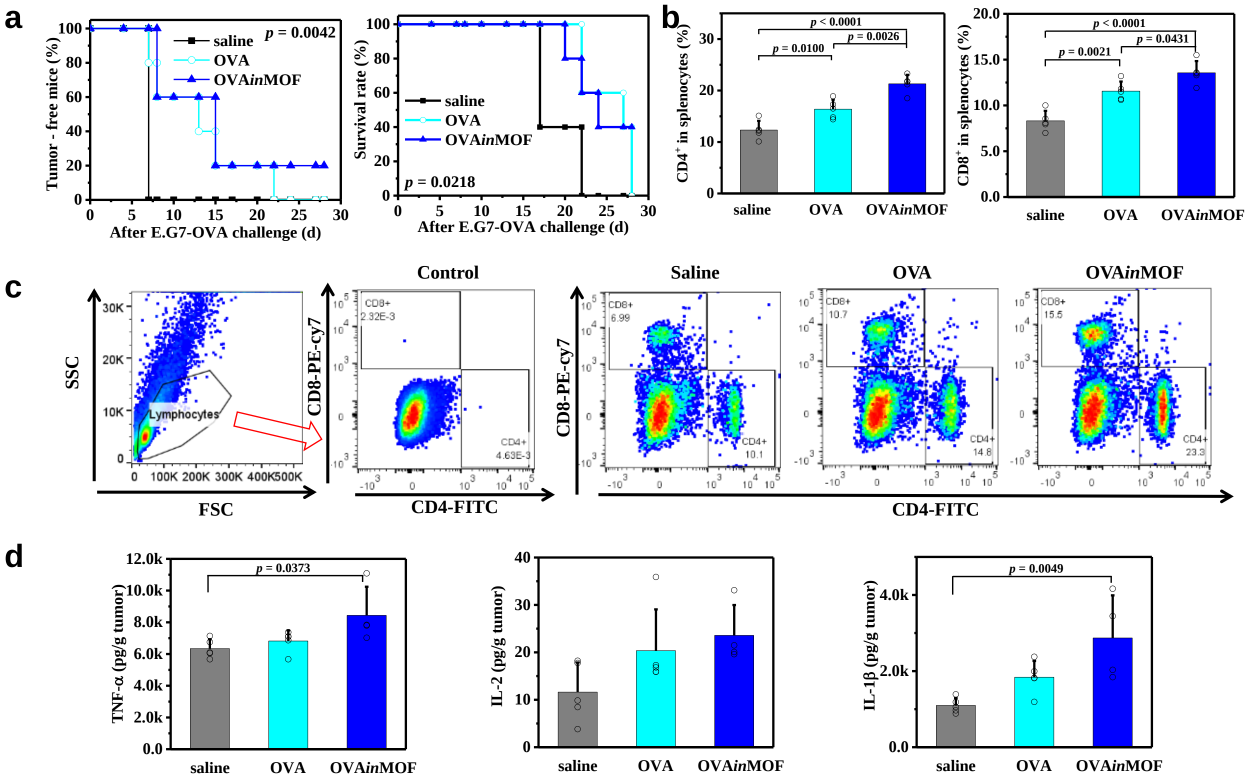

Supplementary Figure 30. OVA-incorporated MOF nanoadjuvants enhance antitumour activity in prophylactic mouse models. **(a)** Prophylactic cancer vaccines consisting of OVA*in*MOF prevent the tumour occurrence and prolong the survival rate of mice. Kaplan-Meier curve of tumour-free mice (a, Left) and mice survival curve (a, Right) after subcutaneously challenging E.G7-OVA cells into the right flank of vaccinated mice (n=5 independent animals, log-rank). **(b, c)** Population of $CD4^+$, $CD8^+$ T cells (b, n=5 independent animals, one-way ANOVA followed by Tukey's multiple comparisons *post hoc* test; $CD4^+$, p<0.0001; $CD8^+$, p<0.0001), gating hierarchy (c, left), control (c, middle) and representative flow cytometry plots of $CD4^+$, $CD8^+$ T cells in splenocytes at the endpoint (c, right). **(d)** TNF-α, IL-2 and IL-1β cytokines content in the tumour sites (saline, OVA, n=5 independent animals; OVA*in*MOF, n=4 independent animals; one-way ANOVA followed by Tukey's multiple comparisons *post hoc* test; TNF-α, p=0.0394; IL-1β, p=0.0064). Data are presented as mean + S.D.

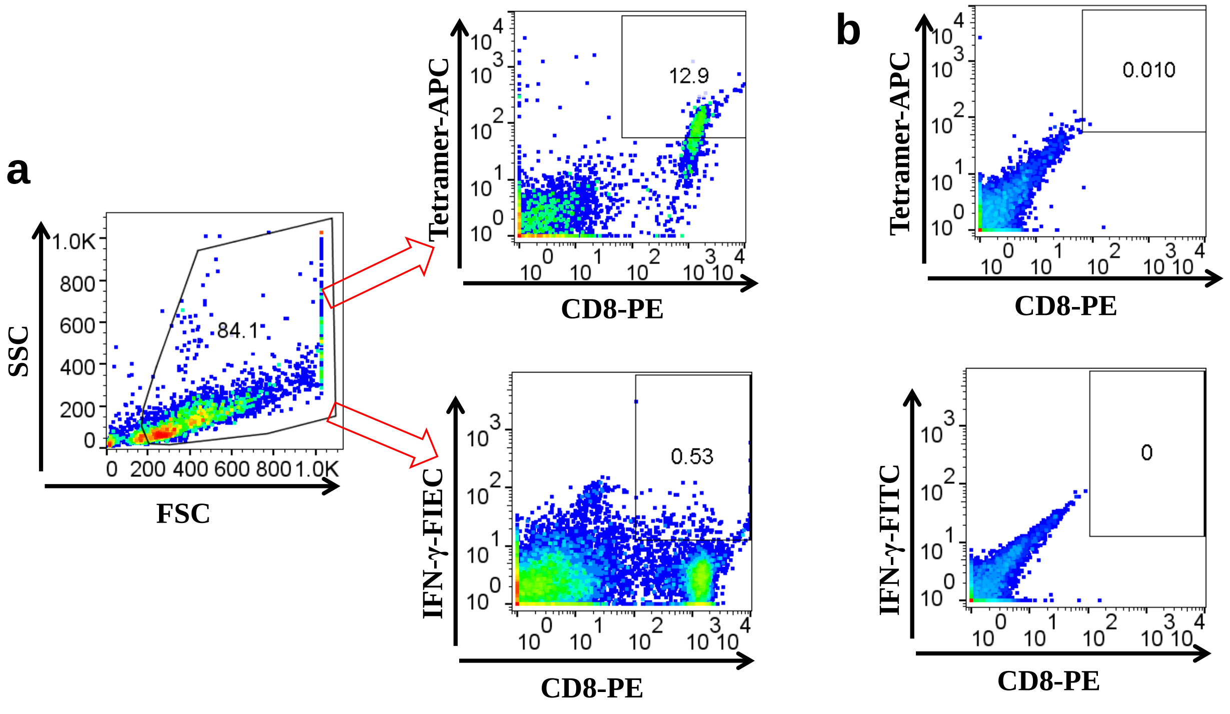

Supplementary Figure 31. (a, b) Gating hierarchy (a) and control dot plots (b) for Figs. 5 e,f and supplementary figure 33 a,b.

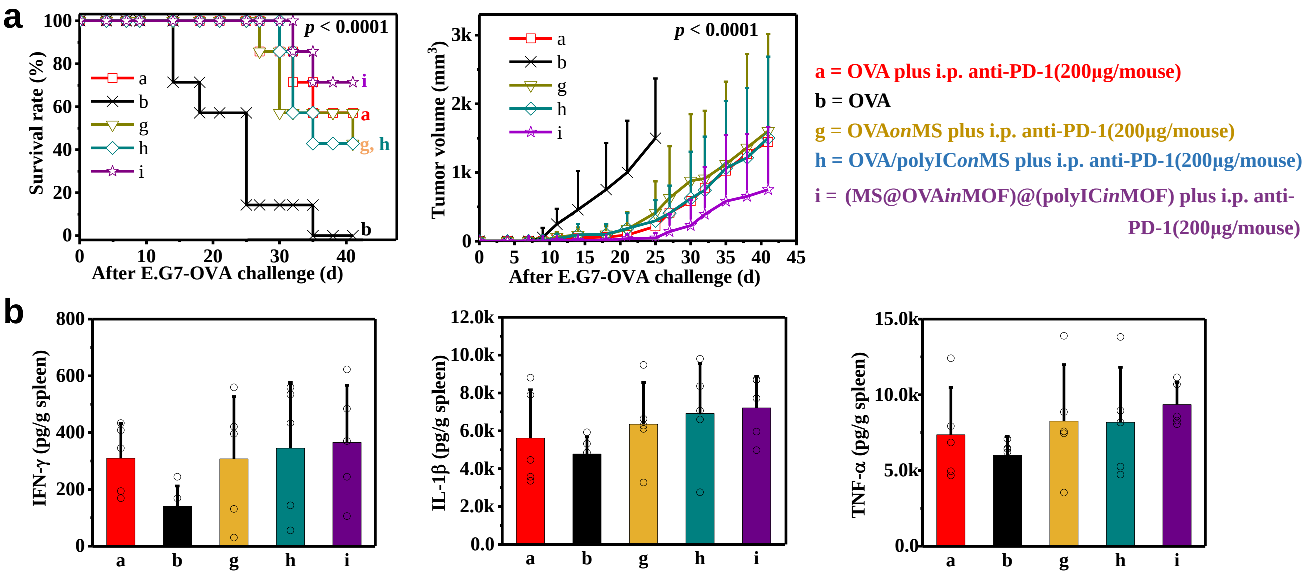

Supplementary Figure 32. (a) Kaplan-Meier curve of mice survival (a, Left) and tumour volume (a, Right) after the same treatment as shown in Fig. 5 c (n=7 independent animals; Left, log-rank; Right, two-way ANOVA). **(b)** Cytokines in spleen at the endpoint (n=5 independent animals, one-way ANOVA followed by Tukey's multiple comparisons *post hoc* test). Data are presented as mean + S.D.

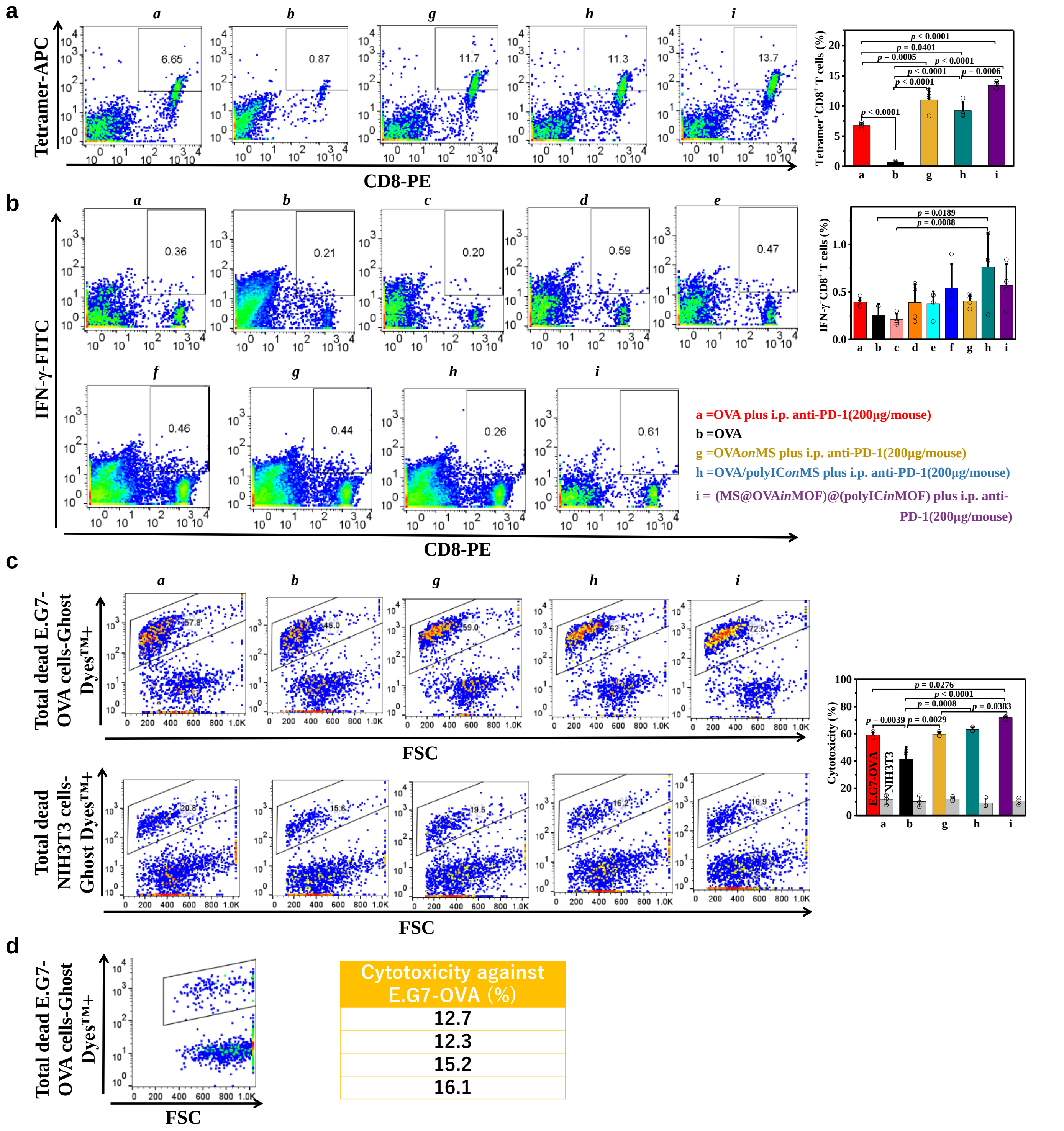

Cytotoxicity against E.G7-OVA (%)
12.7
12.3
15.2
16.1

Supplementary Figure 33. (a, b) Representative flow cytometry plots and population of tetramer^{+}CD8^{+} T cells and IFN-γ^{+}CD8^{+} T cells in splenocytes at the endpoint of treatment as shown in Fig. 5 c (n=4 independent animals, one-way ANOVA followed by Tukey's multiple comparisons *post hoc* test; a, p<0.0001; b, p=0.0118). **(c)** Representative flow cytometry plots and cytotoxicity of the splenocytes derived from different mice against E.G7-OVA cancer cells or healthy NIH3T3 cells (n=3 independent samples, one-way ANOVA followed by Tukey's multiple comparisons *post hoc* test; E.G7-OVA, p<0.0001). **(d)** Representative flow cytometry plots and cytotoxicity of the splenocytes derived from untreated mice against E.G7-OVA cancer cells. All data are presented as mean + S.D.

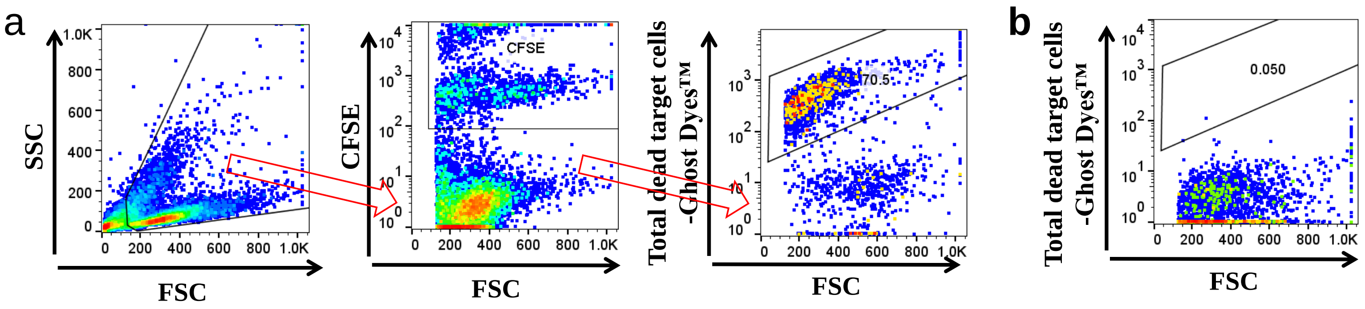

Supplementary Figure 34. (a, b) Gating hierarchy (a) and control dot plots (b) of Figs. 6 c-e and supplementary figure 33 c.

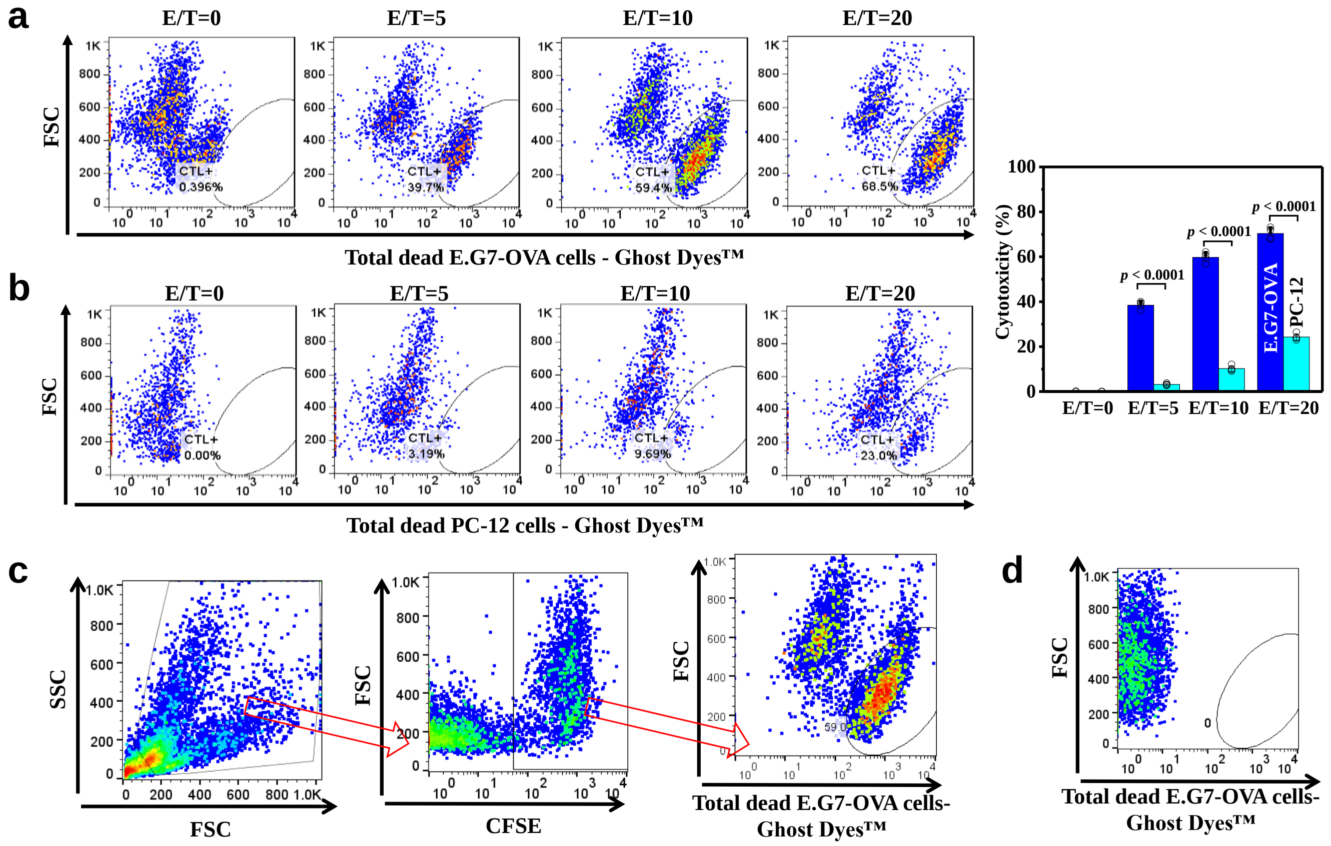

Supplementary Figure 35. (a, b) Representative flow cytometry plots and cytotoxicity of the splenocytes derived from mice in group f of Fig. 5 against E.G7-OVA cancer cells or PC-12 cancer cells at E/T ratio of 0, 5, 10 and 20 (n=4 independent samples; Student's t-test, two-tailed). Data are presented as mean + S.D. **(c, d)** Gating hierarchy (c) and control dot plots of (d).

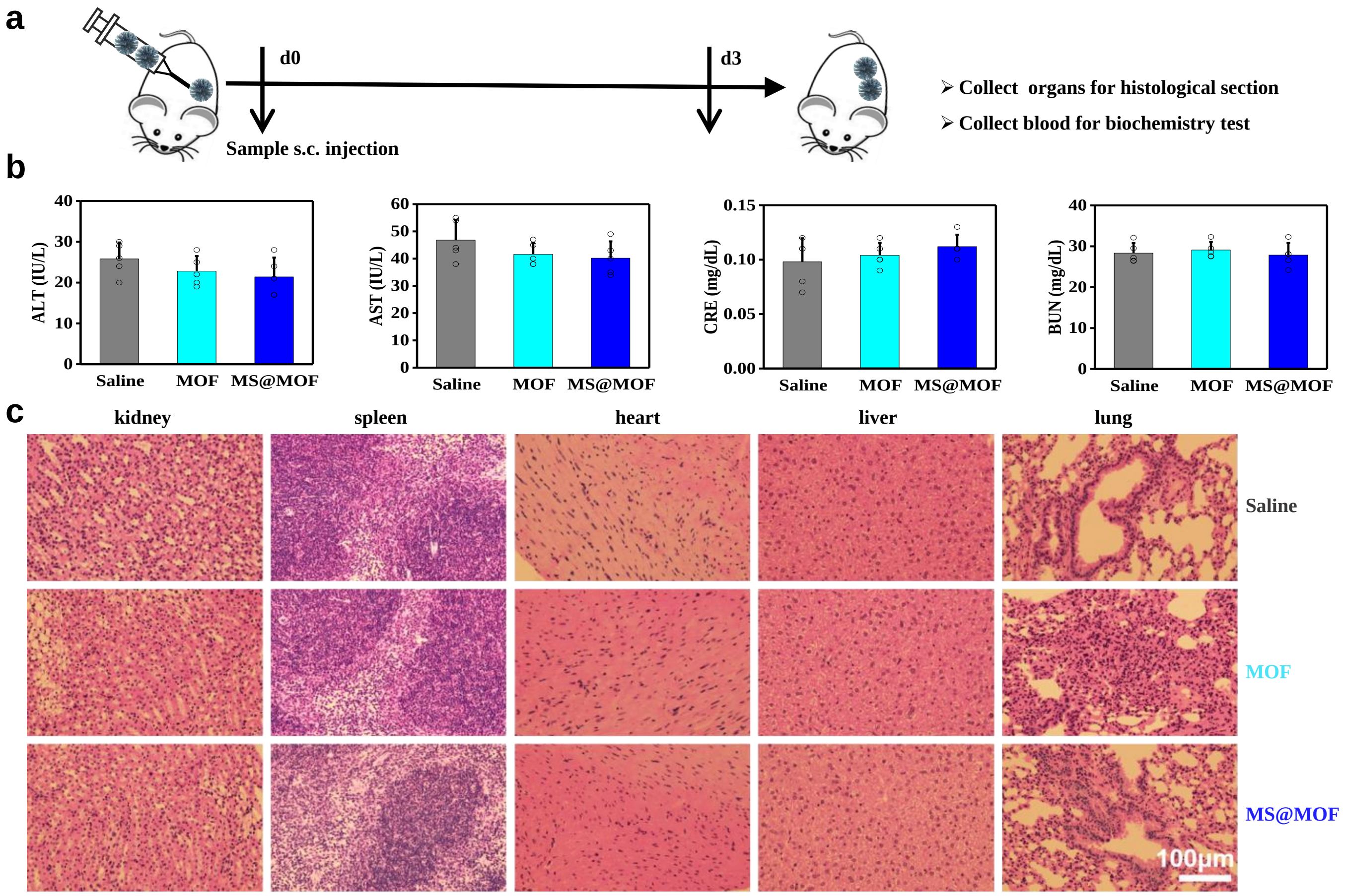

Supplementary Figure 36. Biocompatibility of MOF-gated MS. Healthy C57/BL6 mice were subcutaneously administered with 1 mg of MOF or MOF-gated MS in 100 μL saline per mouse and then the blood biochemistry and tissue compatibility were investigated after three days. **(a)** Timeline of the subcutaneous injection of samples and the collection of organs and blood samples. **(b)** Various biochemistry parameters, such as alanine aminotransferase (ALT), aspartate aminotransferase (AST), creatinine (CRE) and blood urea nitrogen (BUN) (n=5 independent animals, one-way ANOVA followed by Tukey's multiple comparisons *post hoc* test). Data are presented as mean + S.D. **(c)** Histological sections of kidney, spleen, heart, liver and lung after subcutaneous administration of saline, MOF and MOF-gated MS.
