# Supplementary material for: A nanoscale metal organic frameworks-based vaccine synergises with PD-1 blockade to potentiate anti-tumour immunity
Source: Nat Commun. 2020 Jul 31;11:3858. doi: 10.1038/s41467-020-17637-z (PMC7395732; doi:10.1038/s41467-020-17637-z)
Supplement: Supplementary file 2 — Reporting Summary [file 41467_2020_17637_MOESM2_ESM.pdf]

nature research

Corresponding author(s): Xiupeng Wang

Last updated by author(s): Jun 28, 2020

Reporting Summary

Nature Research wishes to improve the reproducibility of the work that we publish. This form provides structure for consistency and transparency in reporting. For further information on Nature Research policies, see Authors & Referees and the Editorial Policy Checklist .

Statistics

For all statistical analyses, confirm that the following items are present in the figure legend, table legend, main text, or Methods section.

n/a	Confirmed	
☐	☒	The exact sample size (n) for each experimental group/condition, given as a discrete number and unit of measurement
☐	☒	A statement on whether measurements were taken from distinct samples or whether the same sample was measured repeatedly
☐	☒	The statistical test(s) used AND whether they are one- or two-sided<br>*Only common tests should be described solely by name; describe more complex techniques in the Methods section.*
☐	☒	A description of all covariates tested
☐	☒	A description of any assumptions or corrections, such as tests of normality and adjustment for multiple comparisons
☐	☒	A full description of the statistical parameters including central tendency (e.g. means) or other basic estimates (e.g. regression coefficient) AND variation (e.g. standard deviation) or associated estimates of uncertainty (e.g. confidence intervals)
☐	☒	For null hypothesis testing, the test statistic (e.g. F, t, r) with confidence intervals, effect sizes, degrees of freedom and P value noted<br>*Give P values as exact values whenever suitable.*
☐	☐	For Bayesian analysis, information on the choice of priors and Markov chain Monte Carlo settings
☐	☒	For hierarchical and complex designs, identification of the appropriate level for tests and full reporting of outcomes
☐	☐	Estimates of effect sizes (e.g. Cohen's d, Pearson's r), indicating how they were calculated

Our web collection on statistics for biologists contains articles on many of the points above.

Software and code

Policy information about availability of computer code

Data collection	FACSAria (BD Biosciences), Living image 4.4.
Data analysis	FlowJo software 10.5.3, ImageJ 1.52g

For manuscripts utilizing custom algorithms or software that are central to the research but not yet described in published literature, software must be made available to editors/reviewers. We strongly encourage code deposition in a community repository (e.g. GitHub). See the Nature Research guidelines for submitting code & software for further information.

Data

Policy information about availability of data

All manuscripts must include a data availability statement. This statement should provide the following information, where applicable:

- Accession codes, unique identifiers, or web links for publicly available datasets
- A list of figures that have associated raw data
- A description of any restrictions on data availability

The data that support the findings of this study are available from the corresponding author upon reasonable request or at a Source Data file.

Field-specific reporting

Please select the one below that is the best fit for your research. If you are not sure, read the appropriate sections before making your selection.

☒ Life sciences ☐ Behavioural & social sciences ☐ Ecological, evolutionary & environmental sciences

For a reference copy of the document with all sections, see nature.com/documents/nr-reporting-summary-flat.pdf

Life sciences study design

All studies must disclose on these points even when the disclosure is negative.

Sample size	The minimum sample size was determined based on previous publication (Nature Biotechnology, 2015; 33: 64-72).
Data exclusions	No data were excluded.
Replication	Sample preparation and characterization were performed at least twice to assure reproducibility. All attempts at replication were successful. In vitro cellular tests and animal experiments were performed twice and by increasing the sample size.
Randomization	All samples, cells and mice were allocated randomly into experimental groups.
Blinding	Investigators were blinded to treatment group allocation. Data were anonymized for analysis.

Reporting for specific materials, systems and methods

We require information from authors about some types of materials, experimental systems and methods used in many studies. Here, indicate whether each material, system or method listed is relevant to your study. If you are not sure if a list item applies to your research, read the appropriate section before selecting a response.

Materials & experimental systems

n/a	Involved in the study	
☐	☒	Antibodies
☐	☒	Eukaryotic cell lines
☒	☐	Palaeontology
☐	☒	Animals and other organisms
☒	☐	Human research participants
☒	☐	Clinical data

Methods

n/a	Involved in the study	
☒	☐	ChIP-seq
☐	☒	Flow cytometry
☒	☐	MRI-based neuroimaging

Antibodies

Antibodies used

anti-CTLA4 (Clone: 9D9, Catalog: BE0164, BioXcell)
anti-PD-1 (Clone: J43, Catalog: BP0033-2, BioXcell)
anti-CD4: BV421 (Clone: GK1.5, Catalog: 100443, BioLegend)
anti-CD8a: PE-cy7 (Clone: 53-6.7, Catalog: 100722, BioLegend)
anti-PD-1: APC (Clone: RMP1-30, Catalog: 109112, BioLegend)
anti-IFN-r: FITC (Clone: XMG1.2, Catalog: 505806, BioLegend)
anti-CD4: FITC (Clone: GK1.5, Catalog: 100405, BioLegend)
anti-CTLA-4: PE (Clone: UC10-4F10-11, Catalog: 561718, BD Biosciences)
anti-SIINFEKL/H-2kb: APC (Clone: eBio25-D1.16, Catalog: 17-5743-82, eBioscience)
anti-CD11b: PE (Clone: M1/70, Catalog: 101207, BioLegend)
anti-CD11c: PE-cy7 (Clone: N418, Catalog: 117318, BioLegend)
anti-CD8a: BV421 (Clone: 53-6.7, Catalog: 100753, BioLegend)
T-Select H-2Kb OVA Tetramer-SIINFEKL-PE (Catalog: TS-5001-1C, MBL)
anti-CD8a: APC (Clone: 53-6.7, Catalog: 100711, BioLegend)
anti-mouse CD80: FITC (Clone: 16-10A1, Catalog: 104705, BioLegend)
anti-mouse CD40: PE (Clone: 3/23, Catalog: 124609, BioLegend)
anti-mouse CD197 (CCR7): PE/Cy7 (Clone: 4B12, Catalog: 120123, BioLegend)
anti-mouse CD16/CD32: (Clone: 93, Catalog: 101302, Biolegend)
anti-mouse MHC II (I-A/I-E): BV421 (Clone: M5/114.15.2, Catalog: 107631, BioLegend)

Validation

Antibody validation was deferred to the manufacturers and was supported by multiple publications.

Eukaryotic cell lines

Policy information about cell lines

Cell line source(s)	E.G7-OVA (ATCC® CRL-2113™) , PC-12 (ATCC® CRL-1721™) and NIH3T3-3-4 (Riken BioResource Center) cells were used.
Authentication	The cell lines authentication was deferred to the manufacturers and was supported by multiple publications. The cell lines were not authenticated by the authors.

Mycoplasma contamination	There was no mycoplasma contamination of the cells.
Commonly misidentified lines (See ICLAC register)	No commonly misidentified cell lines were used.

Animals and other organisms

Policy information about studies involving animals; ARRIVE guidelines recommended for reporting animal research

Laboratory animals	The mice C57BL/6 were purchased from CLEA Inc., Japan. Female mice aged 6-8 weeks were used for experiments. The ambient temperature and humidity were 23±2oC and 50±15%, respectively. The light onset is at 8 o'clock, under a 12h:12h light/dark cycle.
Wild animals	This study did not involve wild animals.
Field-collected samples	This study did not involve field-collected samples.
Ethics oversight	The animal experiment was permitted by the Ethical Committee of the National Institute of Advanced Industrial Science and Technology (AIST), Japan.

Note that full information on the approval of the study protocol must also be provided in the manuscript.

Flow Cytometry

Plots

Confirm that:

- [x] The axis labels state the marker and fluorochrome used (e.g. CD4-FITC).
- [x] The axis scales are clearly visible. Include numbers along axes only for bottom left plot of group (a 'group' is an analysis of identical markers).
- [x] All plots are contour plots with outliers or pseudocolor plots.
- [x] A numerical value for number of cells or percentage (with statistics) is provided.

Methodology

Sample preparation	The sample preparation procedures were described in detail in Methods section.
Instrument	FACSAria flow cytometry
Software	FACSAria (BD Biosciences); FlowJo software 10.5.3
Cell population abundance	1-3 million cells were sorted per sample for staining. 10-50 thousand cells were used for flow cytometry analysis.
Gating strategy	Relevant gating strategies have shown in the main text and Supplementary Information.

- [x] Tick this box to confirm that a figure exemplifying the gating strategy is provided in the Supplementary Information.
